# Non-invasive technology for brain monitoring: definition and meaning of the principal parameters for the International PRactice On TEChnology neuro-moniToring group (I-PROTECT)

**DOI:** 10.1007/s10877-024-01146-1

**Published:** 2024-03-21

**Authors:** Stefano Romagnoli, Francisco A. Lobo, Edoardo Picetti, Frank A. Rasulo, Chiara Robba, Basil Matta

**Affiliations:** 1 Department of Health Science, Section of Anesthesia and Critical Care, Department of Anesthesia and Critical Care, University of Florence, Azienda Ospedaliero-Universitaria Careggi, Florence, Italy; 2 Anesthesiology Institute, Cleveland Clinic Abu Dhabi, Abu Dhabi, UAE; 3 Department of Anesthesia and Intensive Care, Edoardo Picetti, Parma University Hospital, Parma, Italy; 4 Neuroanesthesia and Neurocritical Care Unit, Spedali Civili University affiliated hospital of Brescia, Brescia, Italy; 5 IRCCS Policlinico San Martino, Genova, Italy; 6 Dipartimento di Scienze Chirurgiche Diagnostiche ed Integrate, Università di Genova, Genova, Italy; 7 Consultant in Anaesthesia, Trauma and Critical Care, Cambridge University Hospitals, Cambridge, England; 8 Assistant Professor - University of Cambridge, Cambridge, England; 9 Global Senior Medical Director - Masimo International Irvine, Irvine, CA United States

**Keywords:** Neuromonitoring, Processed EEG, Electroencephalography, TranscranialDoppler, Near infra-red spectroscopy, Pupillometry

## Abstract

Technologies for monitoring organ function are rapidly advancing, aiding physicians in the care of patients in both operating rooms (ORs) and intensive care units (ICUs). Some of these emerging, minimally or non-invasive technologies focus on monitoring brain function and ensuring the integrity of its physiology. Generally, the central nervous system is the least monitored system compared to others, such as the respiratory, cardiovascular, and renal systems, even though it is a primary target in most therapeutic strategies. Frequently, the effects of sedatives, hypnotics, and analgesics are entirely unpredictable, especially in critically ill patients with multiple organ failure. This unpredictability exposes them to the risks of inadequate or excessive sedation/hypnosis, potentially leading to complications and long-term negative outcomes. The International PRactice On TEChnology neuro-moniToring group (I-PROTECT), comprised of experts from various fields of clinical neuromonitoring, presents this document with the aim of reviewing and standardizing the primary non-invasive tools for brain monitoring in anesthesia and intensive care practices. The focus is particularly on standardizing the nomenclature of different parameters generated by these tools. The document addresses processed electroencephalography, continuous/quantitative electroencephalography, brain oxygenation through near-infrared spectroscopy, transcranial Doppler, and automated pupillometry. The clinical utility of the key parameters available in each of these tools is summarized and explained. This comprehensive review was conducted by a panel of experts who deliberated on the included topics until a consensus was reached. Images and tables are utilized to clarify and enhance the understanding of the clinical significance of non-invasive neuromonitoring devices within these medical settings.

**Supplementary Information:**

The online version contains supplementary material available at 10.1007/s10877-024-01146-1.

## Background and methodology

*“Tears come from the heart and not from the brain.”*

(Leonardo da Vinci (1478–1519); The Notebooks of Leonardo Da Vinci)

### Background

Anesthesiologists have historically employed variable combinations of general anesthetic agents and analgesic drugs to induce the reversible state of general anesthesia, characterized by unconsciousness, amnesia, analgesia, and the absence of movement and reflexes. These drugs primarily affect the (mainly central) nervous system, where general anesthetic agents exert their primary effects. Anesthesiologists are considered “masters” in perfusion and tissue oxygenation monitoring. The majority of their practice is dedicated to managing respiratory, cardio-circulatory, and renal functions. Paradoxically, the level of anesthetic impairment of the arousal state and the respective dosages of general anesthetic drugs are guided by indirect hemodynamic parameters, adhering to the Da Vinci anesthetic paradigm mentioned above [1–3].

Indeed, despite the understanding that general anesthetics, sedatives, and analgesics primarily target the central nervous system, monitoring brain function and physiological integrity is rarely conducted outside of neurosurgical operating rooms (OR) and intensive care units (ICU) [1, 2].

Sedatives, such as hypnotics and opioids, are frequently administered to patients with brain injuries (e.g., sepsis-associated neuroinflammation, head trauma, global hypotension-hypoperfusion) and/or those with reduced drug clearance (e.g., liver/renal failure, hypothermia) and/or increased volume of distribution (e.g., fluid overload and tissue edema). In these complex clinical conditions, it is very challenging to predict the action of these drugs on the brain during the clinical course under surgery and anesthesia or in the ICU [4–6]. A global increase in awareness that this plethora of factors could affect the brain under anesthesia and sedation is now leading to a progressive increase in the availability of non-invasive neuromonitoring beyond neuro-anesthesia and neuro-intensive care [7, 8]. Anesthesiologists and critical care physicians are increasingly gaining practice and familiarity with new technologies that explore the physiology and pathophysiology of the central nervous system [8–10].

The purpose of this article is to present to the reader the current nomenclature, meaning, and basic rationale behind non-invasive neuro-monitoring, now widely available in non-neurosurgical ORs and non-neuro-ICUs. The following non-invasive neuro-monitoring technologies will be discussed and presented: (1) processed electroencephalography (pEEG); (2) continuous or quantitative electroencephalography (cEEG, qEEG); (3) near-infrared spectroscopy (NIRS); (4) trans-cranial Doppler (TCD); and (5) pupillometry.

### Methodology

Panel members were selected by the senior author (BM) based on recent scientific literature, membership in scientific societies, and/or invitations as keynote speakers in scientific sessions on the topic of neuromonitoring.

A five-round Delphi process design was utilized. Round one involved presenting a systematic review of the literature, conducted using three electronic databases (PubMed, EMBASE, and the Cochrane library), and made readily available to the panelists at any time. The initial round took the form of a *face-to-face* meeting (mini-Delphi), held at Genoa University Hospital San Martino on December 22, 2023.

After the first round, the panelists presented the list of definitions and principal statements on each topic for the subsequent online Delphi methodology. Following a total of five meetings (from December 2022 to June 2023), consensus was achieved for every definition and meaning. Consensus was considered reached when agreement was higher than 80%.

## Processed encephalography

### Definition

Electroencephalography (EEG)-derived indices and numerical information obtained from the automated analysis of the raw EEG trace.

The use of processed electroencephalography (pEEG) for the management of general anesthesia in operating rooms (ORs) is becoming a standard of care [11]. Expert guidelines and recommendations endorse the use of these tools to prevent both intraoperative awareness during surgery and excessively deep anesthesia [12–14]. Additional emerging applications aim at managing sedation in the ICU to prevent both over-sedation and awareness during paralysis [9, 15].

In addition to raw EEG traces, these systems deliver numerous parameters to assist clinicians in adjusting anesthetics and hypnotics. This section will provide the definition and explanation of each of these parameters.

**Fig. 1:**
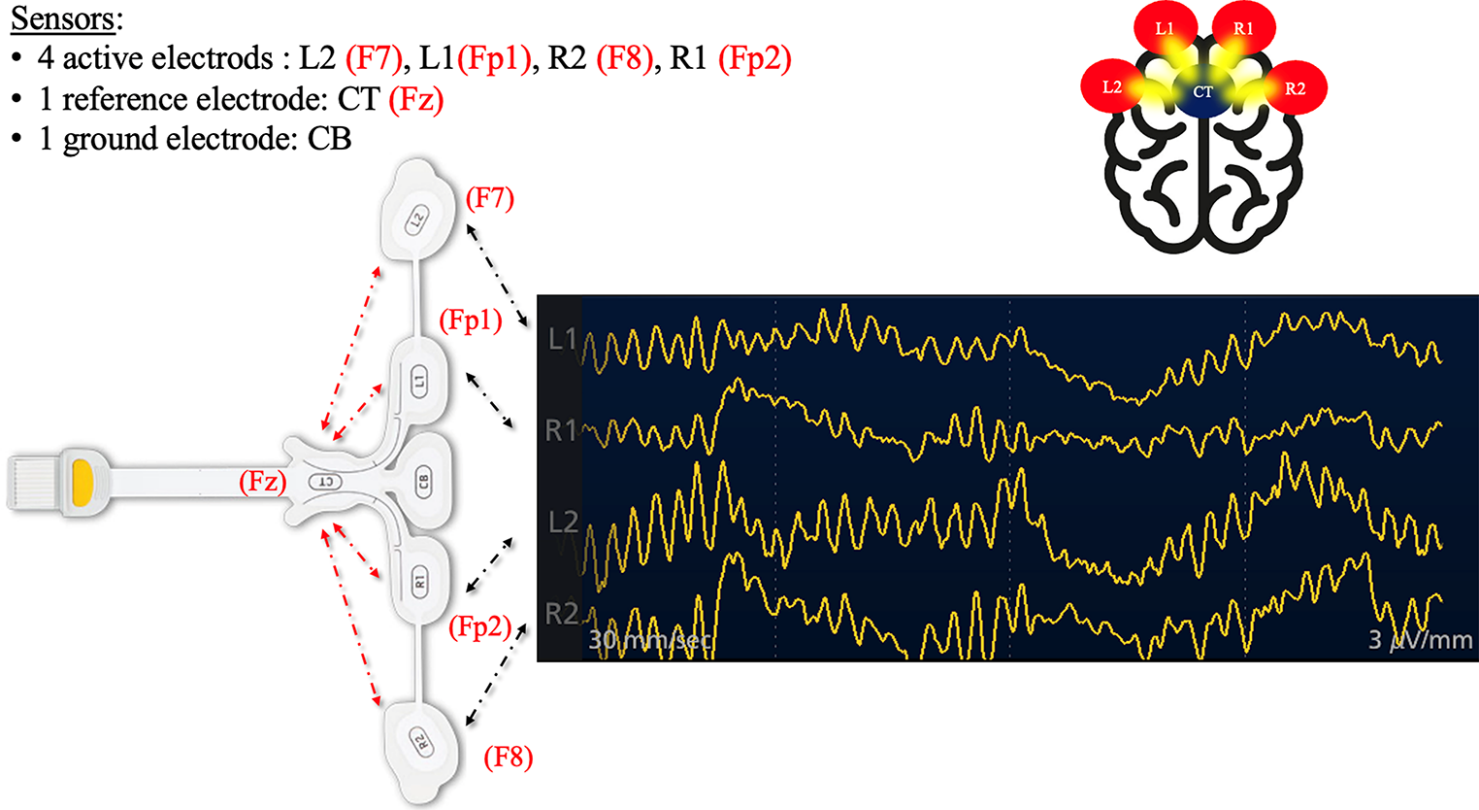
Four-channel pEEG. The pEEG leads are in black, and the corresponding leads of a “standard” EEG are in red [18]. L1: left 1; R1: right 1; L2; left 2; R2: right 2

**Fig. 2:**
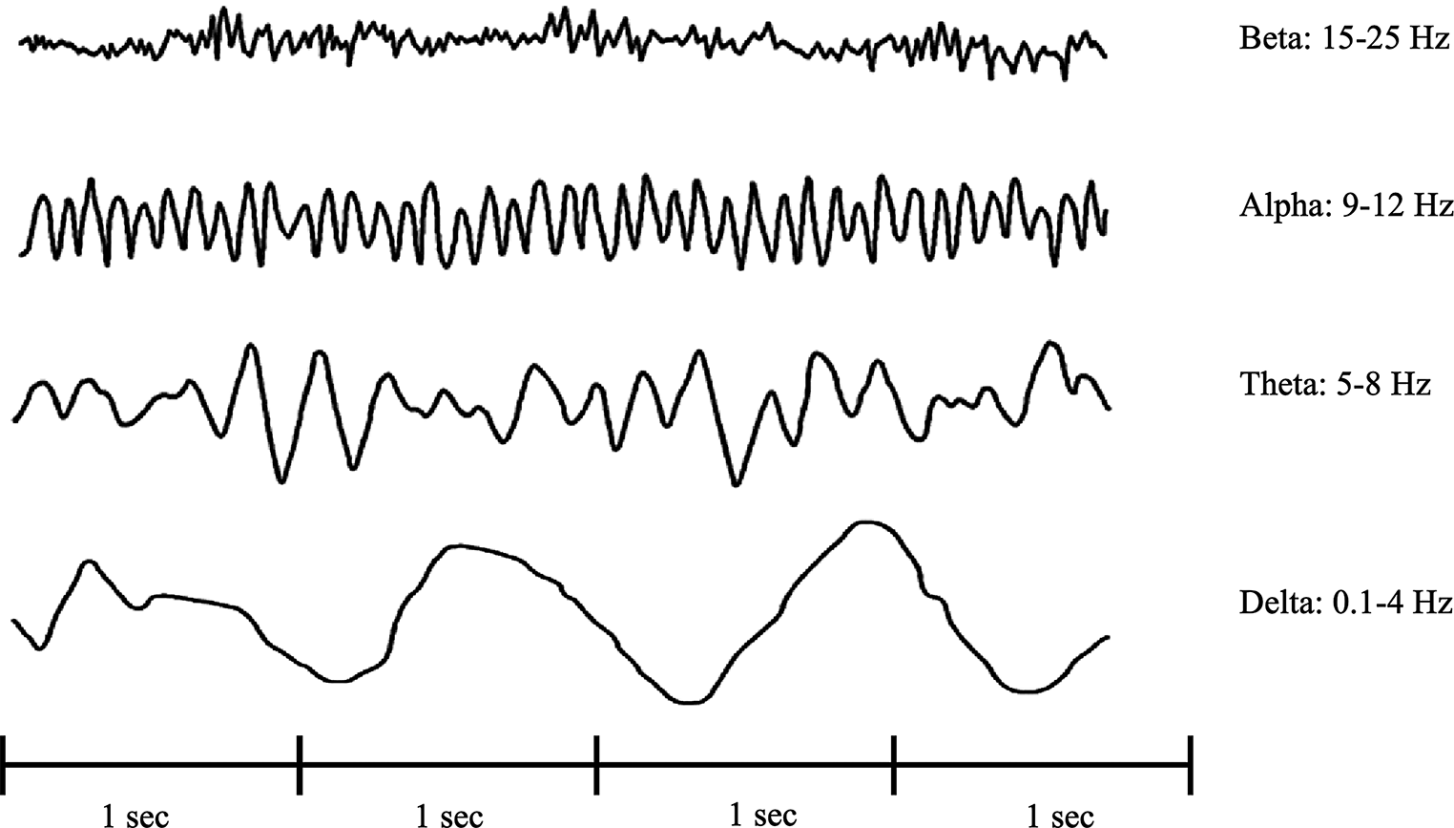
EEG frequencies

**Fig. 3:**
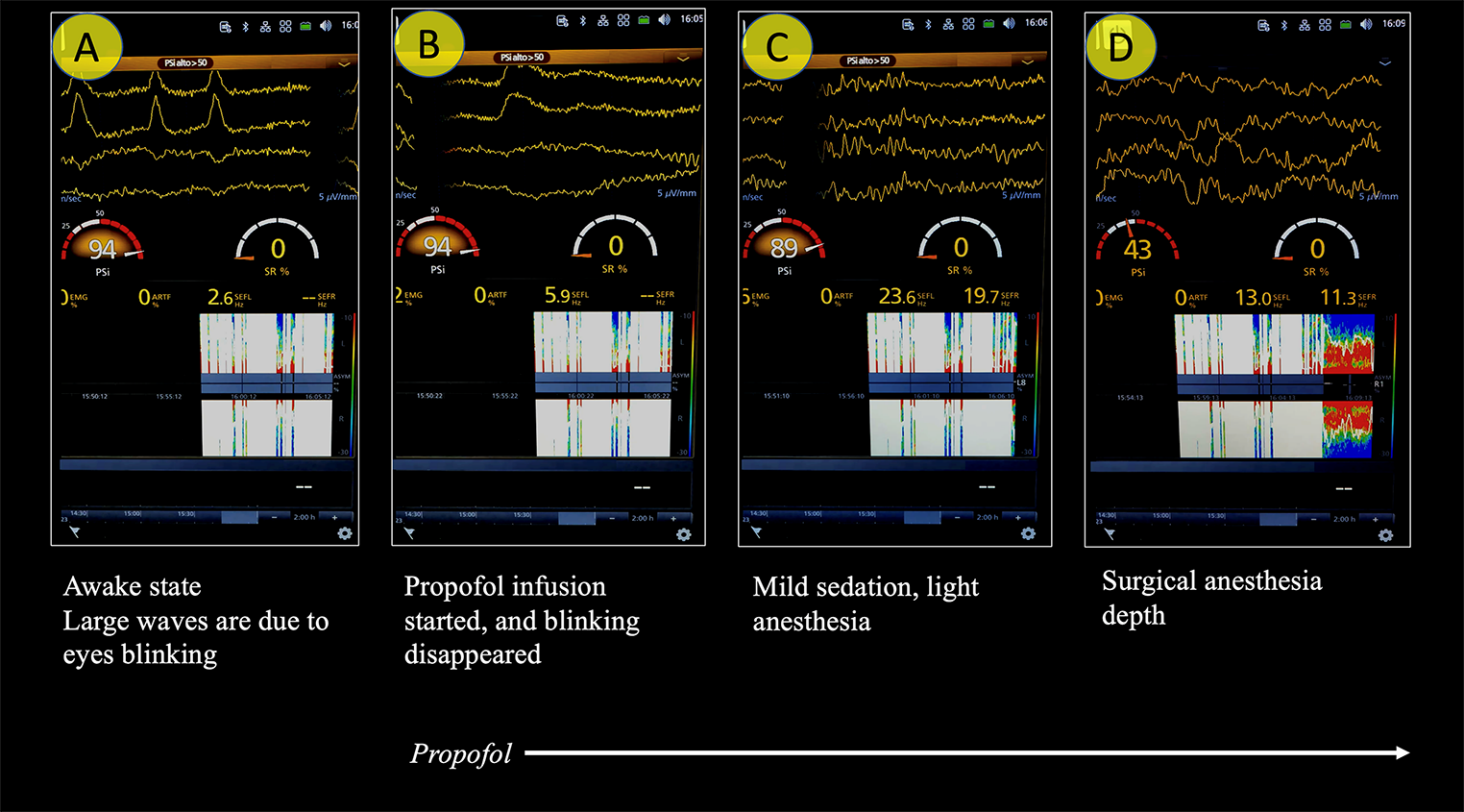
Progressive transition from the awake (A) to the anesthesia state (D). PSi: patient state index; SR: suppression ratio; EMG: electromyography; ARTIF: artifacts; SEFL: spectral edge frequency left side; SEFR: spectral edge frequency right side (see below for details)

**Fig. 4:**
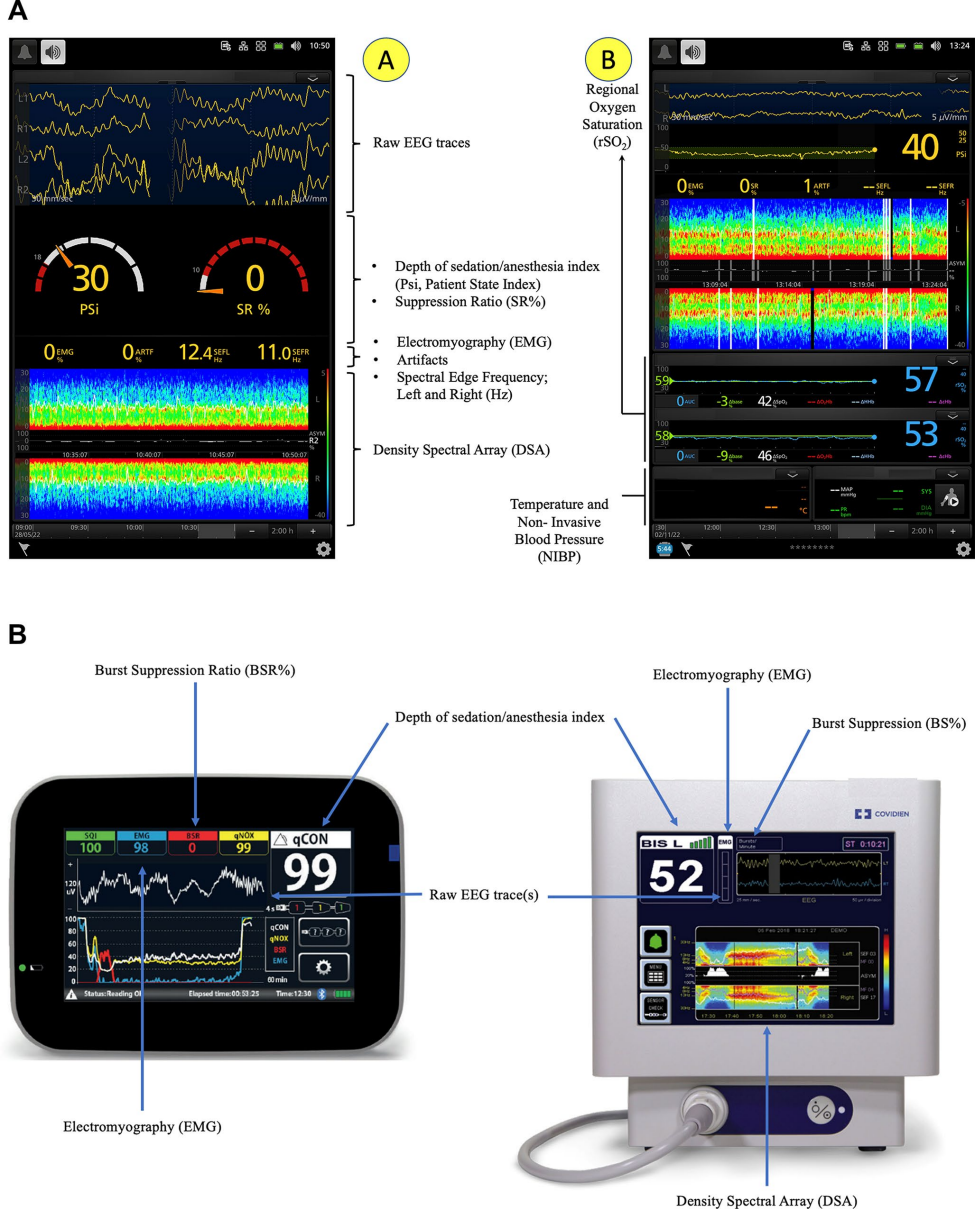
Processed electroencephalography. Configurations without (A) and with (B) O_3_—cerebral oxygen saturation (rSO_2_), see below for details. Processed electroencephalography (other devices)

**Fig. 5:**
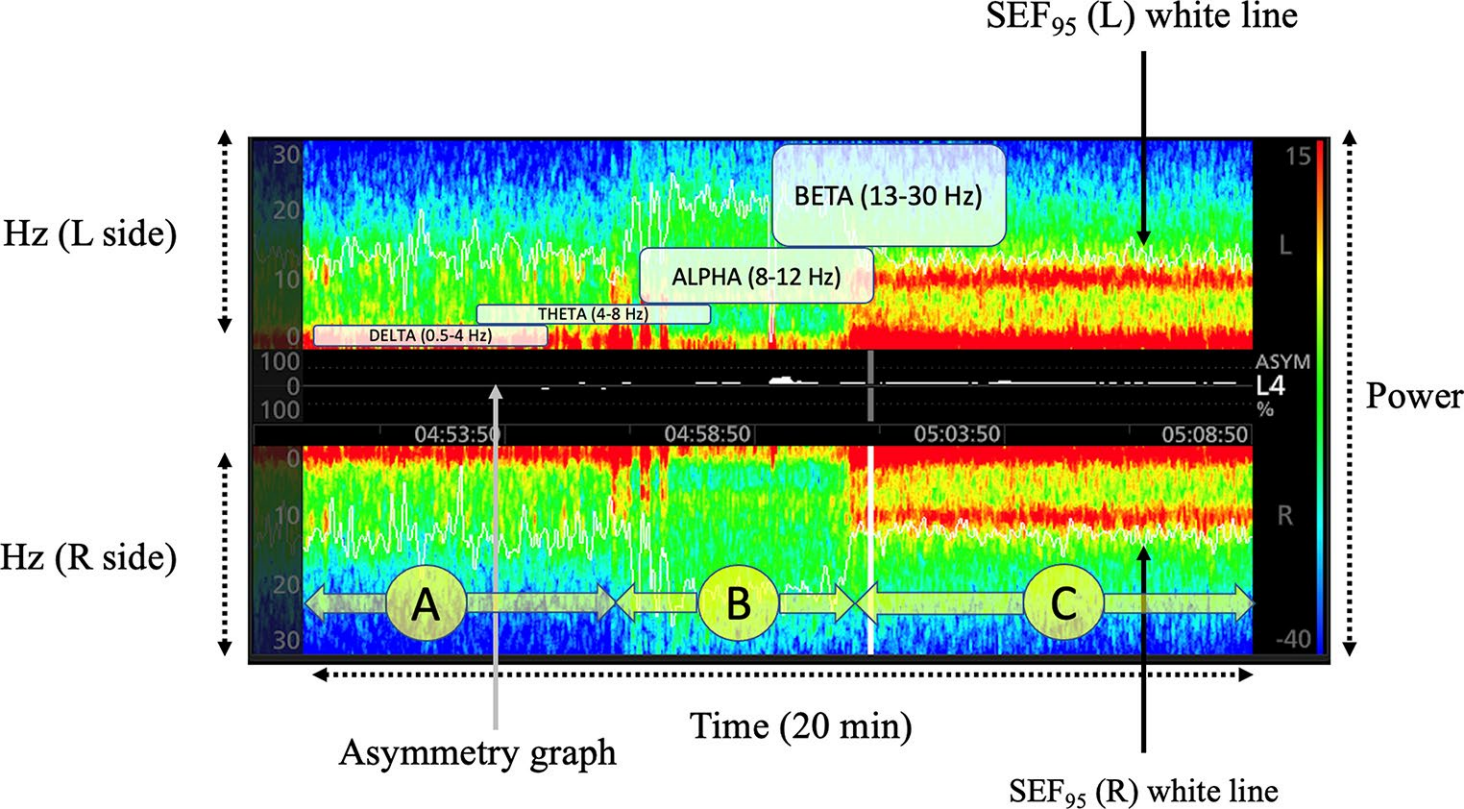
Density Spectral Array. Three different levels of anesthesia can be identified here: (A) strong delta power and weak alpha power; (B) superficial depth of anesthesia with very weak delta and alpha power, and presence of beta activity (note the high level of L and R SEF95); (C) strong delta and alpha power. L: Left; R: Right

**Fig. 6:**
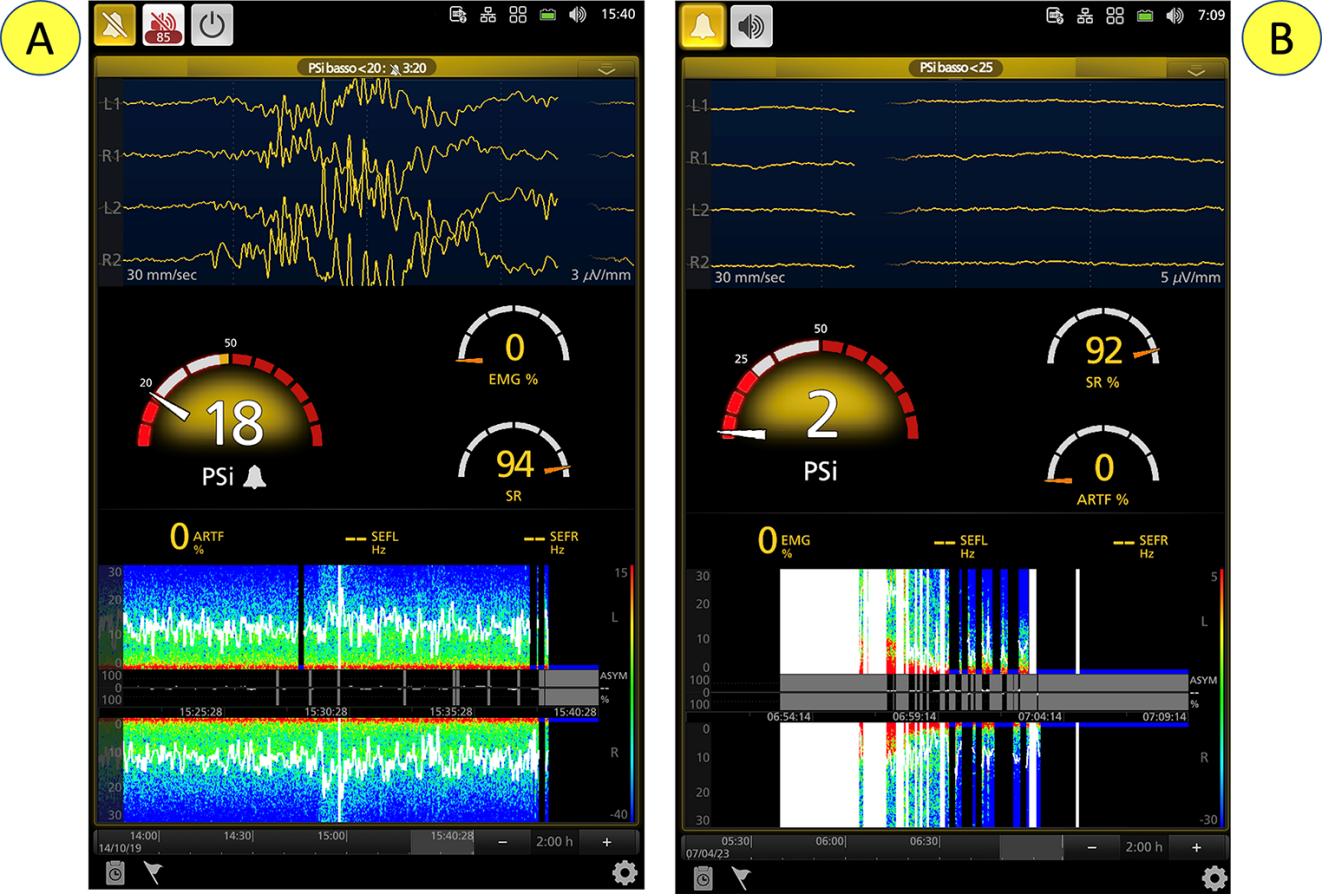
Oversedation. (A) Burst-suppression; (B) total suppression—flat line. Suppression is visible on the DSA as a black vertical line. PSi: patient state index; SR: suppression ratio; EMG: electromyography; ARTIF: artifacts; SEFL: spectral edge frequency left side; SEFR: spectral edge frequency right side

Table 1 and figures 1-6 summarize and show the main parameters displayed by a pEEG monitor.

**Table 1:** Summary of the main parameter displayed by a pEEG monitor [7, 12, 16, 17]

Parameter	Characteristics	Meaning and values
*Raw electroencephalographic (EEG) trace*	Two to four channels (Fig. 1) (depending on the technical characteristics of the monitor) of raw EEG waveforms are displayed in real-time. This includes graphical representations of spatial and temporal variations of electric fields recorded on the skull surface.	A raw EEG trace derived from a combination of basic EEG traces named alpha, beta, gamma, delta, and theta waves (Fig. 2)
*Depth of anesthesia/sedation index*	Dimensionless value calculated by an algorithm (process) applied on the raw EEG traces.	Range 0–100 100: awake state 0: no detectable brain electrical activity 25–50 or 40–60 (depending on the monitor): optimal anesthesia level
*Burst suppression (or burst suppression ratio or suppression ratio) (BS, BRS, SR)*	Pattern of suppression (voltage < 10µV) alternating with higher voltage activity.	Range 0–100%. A value > 0 indicates the presence of burst suppression as a ratio of non-suppressed EEG to suppressed EEG Each monitor has a proprietary algorithm for BS calculation
*Electromyography (EMG)*	Localized or widespread muscular activity on the scalp with an amplitude of 0–10 mV (+ 5 to -5).	Can be displayed as a histogram or as a numerical value in % EMG might interfere with the depth of anesthesia/sedation index level
*Spectral Edge Frequency 95 (SEF*_*95*_ *)*	Frequency value below which 95% of the patient’s total EEG power lies	SEF_95_-L (left frontal lobe) and SEF_95_-R (right frontal lobe) range between 0 and 30 Hz, and correspond to the SEF_95_ recorded in the left and right frontal lobes Might show the “trajectory” of the patient over time
*Density Spectral Array (DSA)*	Quantitative and simplified EEG visualization based on a Fourier transform applied to the EEG signals	Expresses the contribution of each frequency to the global signal, with associated power (in dB) displayed as according to a color scale. The concerned frequencies are displayed on the y-axis, and time on the x-axis The EEG power (-20–40 dB; 10 times the log base 10 of the squared amplitude of a given EEG frequency component) is given for a large spectrum of frequencies (between 0 Hz and 30–40 Hz) Warm colors (red and orange), represent elevated powers and cool colors (blue and light blue) through green, indicate low power

### The raw EEG trace

#### Definition

Graphical representation of unilateral or bilateral frontal brain electrical activity recorded on the scalp surface.

The EEG trace graphically represents the temporal evolution of the sum of the electrical potentials corresponding to the activity of similarly oriented cortical pyramidal neurons. The electrical signal is recorded through sensors that are, for most of the monitors in question here, placed on the frontal (mono- or bilateral) skin surface (Fig. 1). Active, reference, and ground electrode sites are based on the classical (reference method) EEG technology [18].

Raw EEG traces are composed of a coded series of waves primarily characterized by their frequency, measured in cycles per second (Fig. 2).

Combining with each other, the “basic” waves compose characteristic patterns that define the depth of anesthesia or sedation, or the brain electrical activity that is independent of drug effects (e.g., consecutive to neuro-inflammation and hypoperfusion in septic shock) (Figs. 3 and 4a, and 4b). Additional interesting patterns that can be identified on the raw tracing are burst suppression (BS) and full suppression (see below).

### Depth of anesthesia/sedation index

#### Definition

Dimensionless value indicating the depth of anesthesia or sedation and calculated by an algorithm (process) applied on the raw EEG trace.

PEEG monitors, through the application of a proprietary algorithm, analyze and process the raw EEG traces, generating a dimensionless number that represents the depth of sedation and anesthesia [16]. Following the administration of a hypnotic drug, within a delay of approximately 20–30 s (necessary for the machine to analyze a sufficiently long EEG period and dissect the raw trace into a set of basic waves by rapid Fourier analysis or wavelet analysis—see below), the numerical value indicative of hypnosis-anesthesia/sedation depth begins to decrease from a value of 100 (fully awake) to a level appropriate for anesthesia [12, 16] (Figs. 3 and 4a and b).

### Density spectral array

#### Definition

Density spectral array (DSA) is a quantitative and simplified EEG visualization, based on a Fourier transform or wavelet analysis (the EEG is split into its frequency components) applied to the EEG signal. The contribution of each basic frequency to the final signal (power in dB) is expressed according to a color code. The relevant frequencies are displayed on the y-axis, and time is represented on the x-axis (Fig. 4a and b) [12].

Unprocessed EEG observation is a form of time domain analysis, but its reading can be challenging for many clinicians. The DSA is a simplified EEG representation in the frequency domain rather than in the time domain. It uses colors indicating EEG power, commonly expressed in decibels (-20 to 40 dB; 10 times the logarithm base 10 of the squared amplitude of a given EEG frequency component) of a given electroencephalogram frequency component in a large spectrum of frequencies (between 0 Hz and 30–40 Hz) (Fig. 5) [12]. Since the power of an EEG can vary greatly in terms of frequencies, using a logarithmic scale makes it easier to visualize the many frequencies in a single scale. Therefore, the spectrum of a given EEG segment is a plot of power (10 log_10_ (amplitude)^2^) by frequency, as recorded on the left and right side, respectively, in Fig. 5. Using colors, the frequencies that make up the raw EEG traces are represented [12]. Practically, warm colors, such as red and orange, represent elevated powers (in Fig. 4a A, red concerns frequencies in the delta range (< 4 Hz) and alpha range (9–12 Hz), while cool colors, such as blue and light blue through green, indicate low power (scarce presence in the raw EEG)—in Fig. 4a A, this concerns frequencies > 15 Hz. Technically, through the application of a fast Fourier transform, the raw EEG trace is decomposed into its component sinusoids. Once the constituent components of the raw EEG trace have been isolated, they are plotted on a graph, in which the x-axis represents the frequencies and the y-axis represents the amplitude (spectrum of the EEG recording) [12]. The DSA, in turn, is nothing more than the colored representation of the frequency distribution over time (Fig. 5).

### Spectral edge frequency 95 (SEF_95_; L, left; R, right)

#### Definition

Spectral Edge Frequency 95 (SEF_95_) indicates the frequency value below which 95% of the patient’s total EEG power lies. Other thresholds than 95% can also be considered (e.g., SEF50, corresponding to the frequency below which 50% of the EEG power lies) (Figs. 3 and 4a and b).

SEF_95_ determines the frequency below which 95% of the brain activity takes place (frequency where 95% of total EEG power lies below, and 5% above). SEF-L and SEF-R can have values ranging between 0 and 30 Hz as recorded on the left and right frontal lobes, respectively. SEF95 can be used to monitor the anesthesia trend over time. The displayed SEF is clinically useful to track whether spectrogram power is shifting to lower or higher frequencies, allowing for the quick identification of the “trajectory” of the patient over time.

### Burst suppression

#### Definition

BS is a pattern of suppression (voltage amplitude is < 10 µV), alternating with higher voltage activity, with 50–99% of the record containing suppression [17] (Fig. 6). For example, 3 s of bursts followed by 6 s of suppression corresponds to a suppression ratio of 50%.

#### Note

According to the American Clinical Neurophysiology Society’s (ACLS) Standardized Critical Care EEG Terminology [17], “discontinuation” is an alternance of suppression and higher voltage activity, with 10–49% of suppression. This terminology is not used by pEEG devices.

Although the significance of BS is not fully understood, this neuro-physiological phenomenon generally occurs in conditions of sedative/hypnotic drug overdose, septic encephalopathy, hypothermia, and severe brain damage following hemorrhage, hypoxia, and ischemia.

### Electromyography

#### Definition

Identifies localized or widespread muscular activity on the scalp. This activity occurs within a precise frequency range (dependent on the manufacturer) and with amplitudes ranging between 0 and 10 mV (+ 5 to -5 across the 0 line). Note that the EEG signal has an amplitude of about 100 µV (0.1 mV) prior to amplification.

Electromyography (EMG) reflects muscle activity and is not related to cortical activity. It contaminates the EEG signal and should be considered an identifiable disturbance. Due to its influence on the raw tracing, this artifact may artificially increase the value of the depth of sedation index calculated by the machine. Notably, high EMG activity may indicate patient arousal following noxious stimulation or a lack of muscle relaxation (Figs. 3 and 4a, and 4b).

### Asymmetry

#### Definition

Unequal electrical activity between the right and left hemispheres, either regarding voltage and/or frequency.

Many cerebral diseases might be associated with an asymmetry of electrical activity (e.g., tumor, single-site hypoperfusion, intracranial bleeding, seizures). The asymmetry graph (Fig. 5) illustrates the difference in brain activity between the left and right sides based on a measurement of asymmetry, ASYM, displayed between the right and left DSA graphs. Clearly, the asymmetry graph can only be obtained when a bilateral sensor is used (also shown in Fig. 4aB and 6).

## Qeeg: the quantitative continuous electroencephalogram

### Definition

Numerical analysis and/or visual transformations of long-lasting raw EEG signals.

EEG is a continuous and non-invasive monitoring of cerebral function, recording the electrical signal generated by the pyramidal neurons of the brain cortex [19, 20]. Quantitative EEG (qEEG), utilizing algorithms to transform and compress the raw EEG signal, allows rapid screening and display of large amounts of data using a graphical representation [19–21]. This way, some information is simplified to facilitate interpretation, even by non-neurophysiologists [19–21]. In the ICU, this allows for real-time guidance of treatment [19]. However, as recommended by the American Clinical Neurophysiology Society, continuous EEG in the ICU should be frequently (at a minimum twice daily) reviewed by trained personnel for technical quality and in case of important changes [22, 23]. Early identification and treatment of these changes in brain function are of paramount importance to improve the outcome of patients. Recognized indications for the utilization of continuous EEG in the ICU include [23]:

- NCSs and other paroxysmal events detection.
- Effectiveness of anti-seizure therapy assessment.
- Cerebral ischemia identification.
- Sedation/metabolic suppression monitoring.
- Ischemic encephalopathy prognostication.

Different qEEG monitors are available and able to generate several graphs/trends, allowing ICU physicians to rapidly screen long periods of EEG to detect changes over time [19]. Commonly used trends include [19–21]:

- Color density spectral array (CDSA).
- Asymmetry index.
- Power ratio.
- Suppression ratio.
- Amplitude EEG.
- Envelope trend analysis.

Of note, several of the parameters provided by qEEG monitors are similar to those provided by pEEG monitors.

### Color density spectral array

#### Definitions

CDSA is a three-dimensional, EEG frequency-based graphical display with time reported on the x-axis and frequency on the y-axis [20]. Specifically, spectrograms utilize an algorithm (FFT) to process the raw EEG signal and display the power of the identified frequencies (y-axis) according to a color code (z-axis) over time (x-axis) [20]. Colors may vary according to the manufacturer [19, 20].

**Fig. 7:**
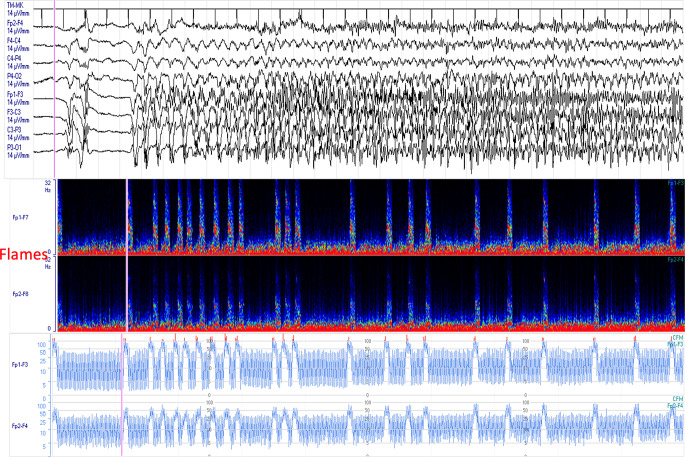
– Seizures detection by cEEG. Flames are evident (bottom side)

CDSA is generally utilized for seizure detection [21–24] (Fig. 7). Seizures are associated with an increase in frequency and amplitude of the EEG. This manifests on the CDSA trend as a paroxysmal event with increased power (i.e., a “solid flame” pattern presenting as an abrupt onset of higher EEG power with a smooth edge similar to candlelight) [19–21].

### Asymmetry index

#### Definition

This method compares the power of a frequency band between hemispheres [20, 21]. The provided index may be expressed as an absolute number (i.e., the higher the number, the greater the asymmetry) or as a relative value (i.e., a positive or negative value differentiates between higher power on one side as compared to the other) [21].

In a spectrogram (frequency range displayed on the y-axis), the utilized color may indicate the side with more power at a given frequency, and the color intensity represents the asymmetry level [20, 21]. The asymmetry index is particularly useful for the detection of focal seizures [21, 23].

### Power ratio

#### Definition

The ratio between the power of two selected frequency bands.

Detailed information regarding the power spectrum can be obtained by plotting the power of specific frequency bands (i.e., the area under the power spectrum curve) such as the alpha (8–13 Hz) and the delta (< 4 Hz) frequency bands [21]. The alpha/delta power ratio is frequently utilized [19, 21]. A reduction in faster frequencies and an increase in slower frequencies are observed in the case of cerebral hypoperfusion and ischemia [19]. Thus, the progressive decline of the alpha/delta ratio, observed in this scenario, can be considered a useful tool for detecting delayed cerebral ischemia (DCI) after subarachnoid hemorrhage (SAH) [19, 23].

### Burst suppression ratio

#### Definition

The burst suppression ratio (BSR) represents the percentage of time where the EEG is suppressed in a given epoch (i.e., 3 s suppression and 1 s of burst corresponds to a BSR of 75%) [21].

The BSR allows assessing the depth of the alteration of wakefulness during refractory status epilepticus or other conditions (i.e., barbiturate coma for refractory intracranial hypertension), where the goal is to induce a burst suppression pattern in the EEG [23]. BSR can also be useful for cardiac arrest neuro-prognostication [21].

### Amplitude eeg

#### Definition

The raw EEG, after being filtered to a frequency range of interest, is displayed on a compressed time scale [21]. For each epoch (i.e., 1–2 s), the maximum and minimum amplitudes are plotted and connected by a vertical line [21].

Amplitude EEG is one of the earliest forms of qEEG analysis, mainly utilized in neonatology, where only 2–4 electrodes are generally utilized (P3-P4 in the single channel system, and C3-P3/C4-P4 in a dual channel system) [21]. Amplitude EEG has been utilized for seizure detection (sudden increase in voltage) [21].

### Envelope trend analysis

#### Definition

The raw EEG, similar to the Amplitude EEG, is filtered to a specified frequency range with the median waveforms amplitude plotted for a given epoch (i.e., 10–20 s) [21]. This modality has been frequently utilized in neonates for seizure detection [21].

Table 2 summarizes the commonly used qEEG trends in the ICU with possible clinical applications.

**Table 2:** Commonly used qEEG trends in the ICU with possible clinical applications

qEEG TRENDS	CLINICAL USE
CDSA (CSA, FFT, DSA)	Seizures detection
Asymmetry index	Detection of seizure lateralization
Power ratio (alpha/delta)	DCI detection in SAH
BSR	Prognostication of CA Depth of sedation monitoring during the management of Refractory Status Epilepticus or refractory intracranial hypertension
AEEG	Seizure detection
Envelope trend analysis	Seizure detection

**Abbreviations**: qEEG = quantitative electroencephalography, CDSA = color density spectral array, CSA = color spectral array, FFT = fast Fourier transform, DSA = density spectral array, DCI = delayed cerebral ischemia, SAH = subarachnoid hemorrhage, BSR = burst suppression ratio, RSE = refractory status epilepticus, AEEG = amplitude electroencephalography, CA = cardiac arrest

## Near-infrared spectroscopy for the non-invasive monitoring of somatic and cerebral regional oxygen saturation (rSo_2_)

### Definition of near infra-red spectroscopy

Near infra-red spectroscopy (NIRS) is a noninvasive, real-time measure of tissue oxygen saturation (rSo_2_) that estimates the saturation of hemoglobin in oxygen [25].

### Definition of cerebral NIRS

NIRS is applied to cerebral tissue (frontal lobes) to estimate the oxygen saturation of the cortical layer of the brain.

### NIRS technology

Based on the Beer-Lambert Law (modified) for a scattering medium, NIRS is used to measure the concentration of different “chromophores” (i.e., biological molecules that absorb electromagnetic radiation [EMR]), according to the following [24, 25]:

OD_λ_ = ελ • L • c • DPF + OD_R,λ_

OD_λ_ = optical density of a medium.

ελ = extinction coefficient of a chromophore.

L = source-detector separation (wavelength-dependent).

c = concentration of the chromophore.

DPF (differential pathlength factor) = correction factor designed to estimate how far a photon travels through the tissue. It is dependent on absorption and scattering coefficients of the tissue.

OD_R,λ_ = geometry and wavelength-dependent correction factor.

A matrix algebra, consisting of different equations (one for each wavelength), is elaborated to quantify the concentration of multiple chromophores (e.g., oxyhemoglobin, O_2_Hb, and deoxyhemoglobin, HHb) [25].

**Fig. 8:**
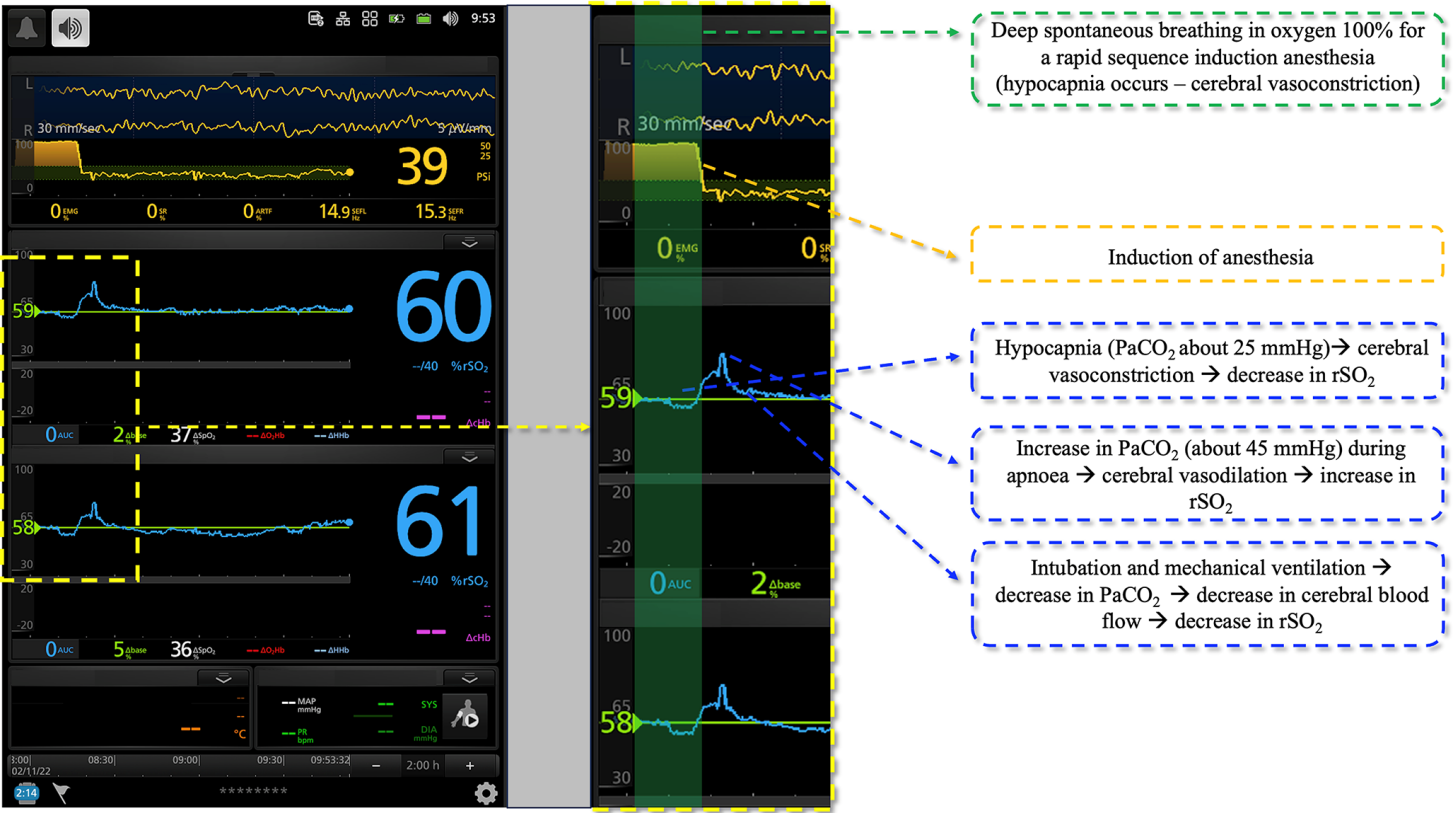
The case of a rapid sequence induction of anesthesia. The oscillations in cerebral saturation are related to changes in cerebral blood flow due to changes in PaCO_2_. The effects of the changes in PaCO_2_ are detailed on the right side of the figure. %rSO_2_: regional oxygen saturation of the frontal cerebral lobes (both sides)

Cerebral oximetry measurements are expressed in percentage. Of note, changes in rSO_2_ relative to a baseline initial identification (i.e., pre-induction of anesthesia) offer useful clinical information (Fig. 8).

EMR is diffused from the light source to the neurons and brain tissue. Photons reach the brain by traveling through different tissues (i.e., skin, skull, muscle, fat, and meninges). Photon detectors (e.g., photodiodes) are located at 2–4 cm from the light source. To separate the contribution to absorbance of non-brain tissues chromophores from the contribution of brain chromophores, the stronger signal (proximal and with a low level of absorbance) is subtracted from the deeper level of absorbance. Since both signals are affected (equally) by non-brain tissues, the difference between the two signals is due to absorbance through the brain [25].

Brain rSO_2_ derives from arterial, capillary, and venous blood combined. Since the relative arterial-to-venous ratio is fixed (e.g., 30:70 or 25:75, depending on the device), the oxygenation of venous blood leaving the cerebral capillary beds can be estimated using a simple equation:

Venous Cerebral O_2_ = (Tissue O_2_ – 0.3 (or 0.25) X 100%)/0.7 (or 0.75).

Of note, even if commercially available cerebral oximeters assume a fixed arterial-to-venous ratio, the true percentage of venous blood can range from 33 to 84%, and the arterial-to-venous ratio may change in many clinical conditions [25].

Two sensors are needed to measure the concentrations of O_2_Hb, HHb, and the total hemoglobin (tHb) in the frontal cortex of the brain.

**Fig. 9:**
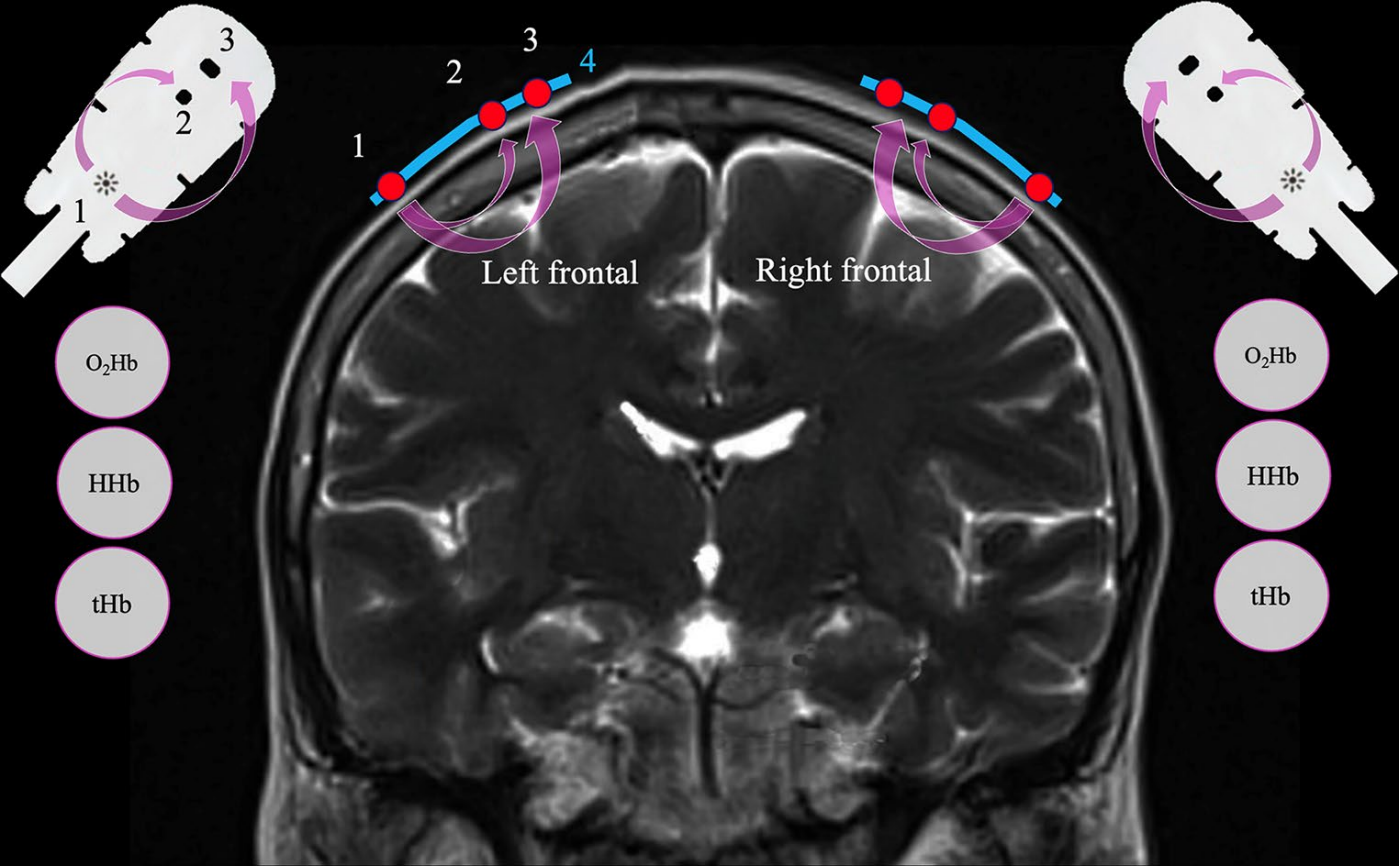
NIRS photodetectors for the estimation of O_2_Hb, HHb, and tHb. The probes with their photon detectors are placed 2–4 cm away from the light source. 1: Incident light source [light-emitting diode (LED)]; 2–3: Proximal (superficial) and distal (deep) photodetectors; 4: Adhesive NIRS probe

Photons travel from an emitter light source (light-emitting diode; LED) to both sensors. The course of the photons depends on the distance between the LED and the sensors, and the course directed to the distal sensor passes deeper through the brain (Fig. 9), whereas the path to the proximal sensor is more superficial. The difference between the two allows for the mathematical removal of the contribution of extracranial tissue to the calculation of rSO_2_ [26, 27].

Cerebral (frontal cortex) rSO_2_ is a global indicator of cerebral perfusion that reflects the balance between oxygen delivery and consumption. The measurement of cerebral Hb-oxygen saturation by means of NIRS is associated with important clinical outcomes [28] as it represents the proportion of O_2_Hb to tHb in the cerebral vasculature. Many clinical conditions such as hypotension and/or hypoperfusion, ischemia (caused by embolism), compression, or impairment of venous drainage may affect cerebral oxygenation, and in most of these situations, arterial Hb oxygen saturation (SaO_2_) may remain within the normal limits. By examining rSO_2_ and its sub-components (O_2_Hb, HHb, and tHb), the clinician can identify episodes of cerebral desaturation and intervene according to algorithms that have shown to successfully help improve cerebral oxygenation [28–30].

## Transcranial Doppler (see the appendix)

### Definition

**Fig. 10:**
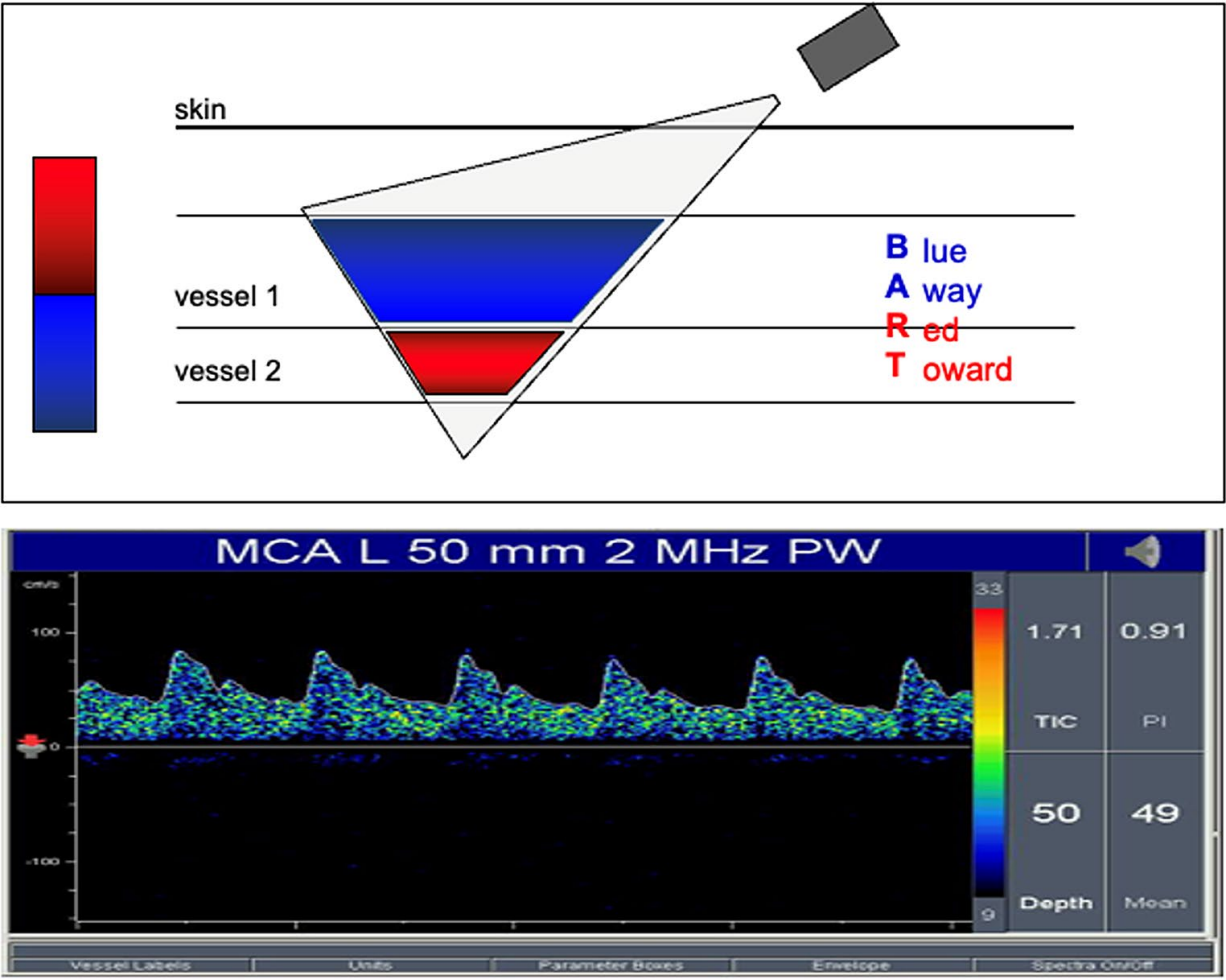
TCD flow velocities waveform. MCA: middle cerebral artery; MHz: mega Hertz; PW: pulse wave

Transcranial Doppler (TCD) is a noninvasive ultrasound (US) tool, which allows the measurement of cerebral blood flow velocity (CBF-V) in the major intracranial arteries using a low-frequency (≤ 2 MHz) Ultra Sound (US) wave (Fig. 10).

### Transcranial color-coded duplex Doppler sonography

#### Definition

**Fig. 11:**
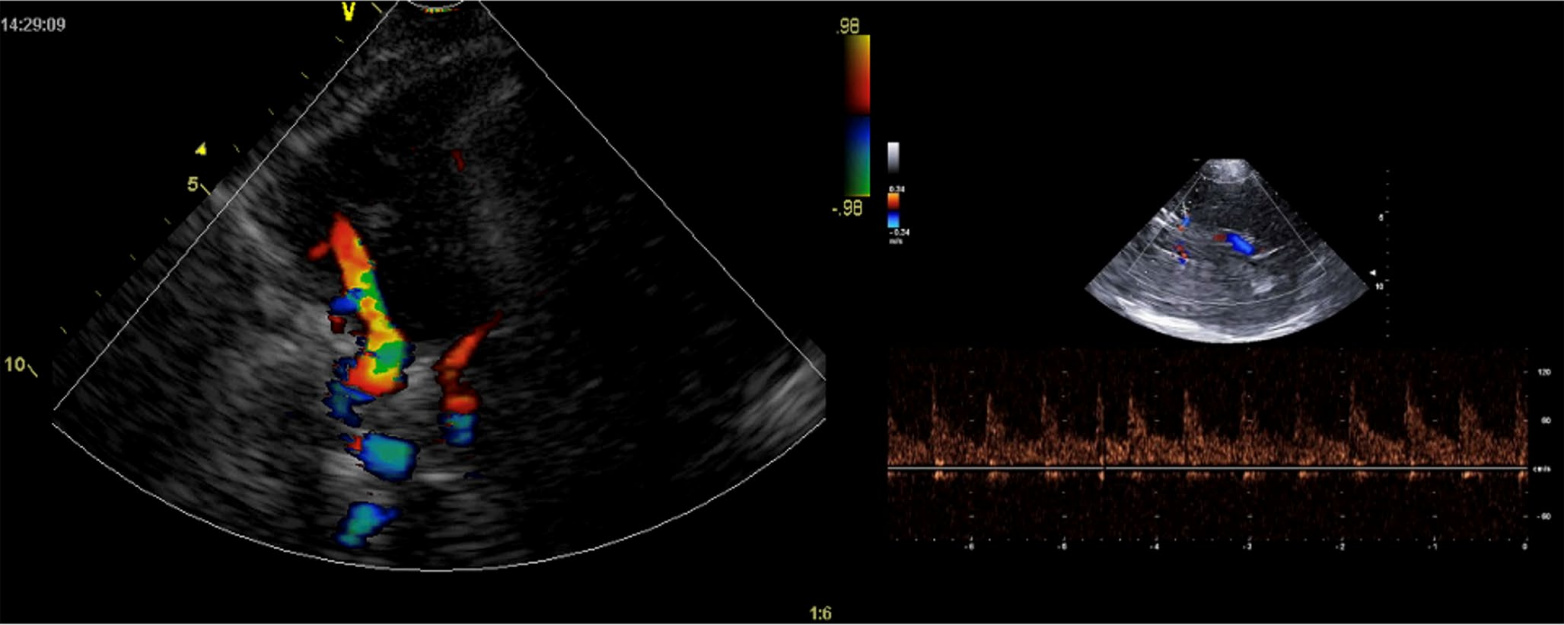
TCCD, color and B-mode. TCCD combines B-mode with Doppler pulse wave technology. The B-mode imaging shows an image of the skull, brain, and blood vessels. Once the desired blood vessel is found, it is shown with the color function

Transcranial color-duplex Doppler sonography (TCCD) is a technology that combines Doppler pulse wave technology with B-mode, which is the 2-dimensional imaging of intraparenchymal structures (Fig. 11).

TCCD requires the same transducers and equipment as focused echocardiography (cardiac 3–5 MHz probe) [31, 32]. Using TCCD is very helpful in various settings, such as in acute ischemic stroke, for the non-invasive estimation of intracranial pressure (ICP), and for the identification of a brain midline shift after intracranial hemorrhage (ICH) or due to other intracranial lesions (tumor, abscess, advanced brain edema). Other applications of the technology include ICP and CBF monitoring in traumatic brain injury (TBI), and the clinical diagnosis of brain death [31–34].

### Traditional windows for TCD/TCCD examination

#### The transtemporal window

**Fig. 12:**
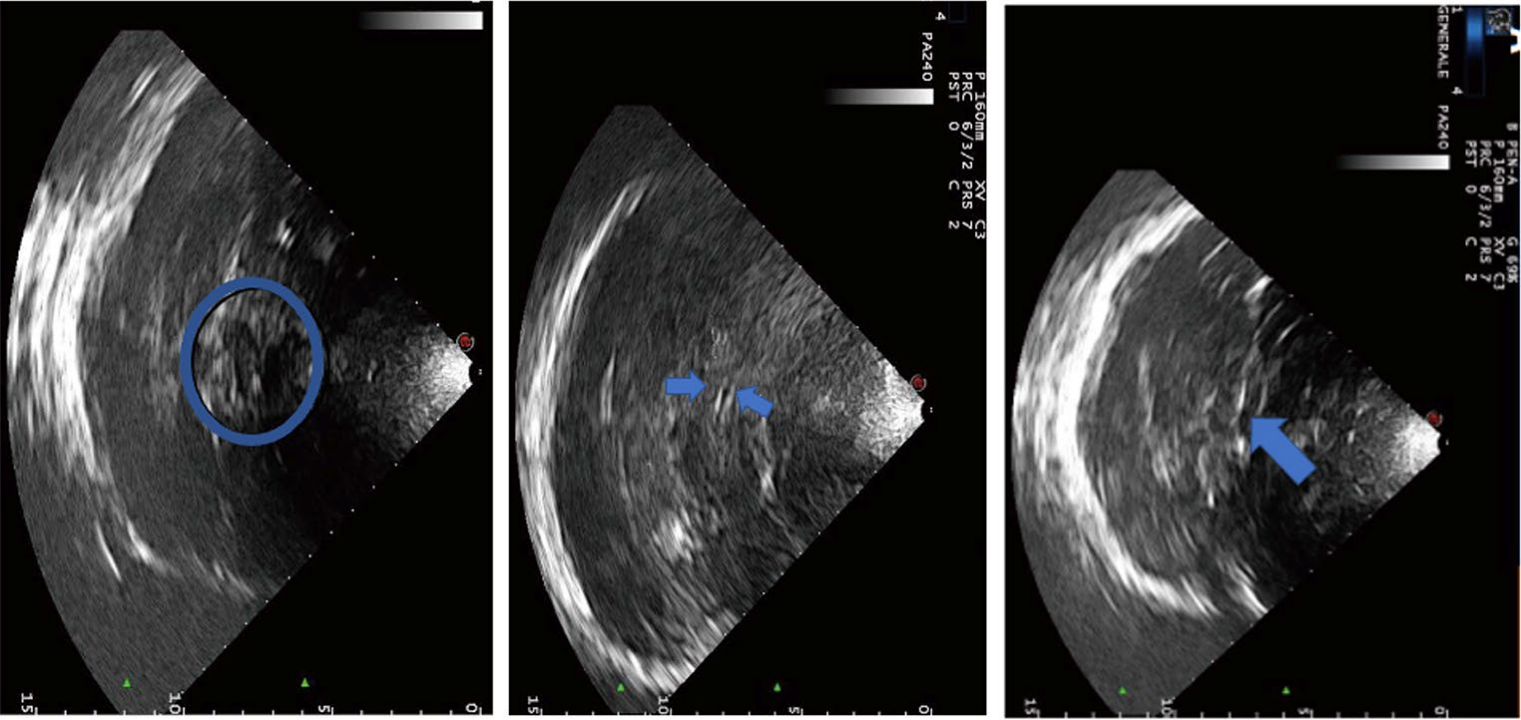
Mesencephalic (left panel), diencephalic (central panel), and ventricular plane (right panel). Blue indicators highlight the butterfly-shaped mesencephalus, and the third and the lateral ventricles, respectively

The main landmark for probe position is in the superior part of the zygomatic arch and nasal to the pinna of the ear [35, 36]. This window allows the inspection of the terminal internal carotid artery (ICA), middle cerebral artery (MCA), anterior cerebral artery (ACA), posterior cerebral artery (PCA), and communicating arteries (Fig. 12).

The mesencephalic plane initially identifies the ipsilateral/contralateral temporal bones and the third ventricle (midline structures). Then, the butterfly-shaped hypoechoic cerebral peduncles surrounded by hyperechoic star-shaped basal cisterns are visualized, forming an overall heart-shaped form.

Anterior angulation of the probe with a depth of 6–7 cm allows the insonation of the ACA and the A1-precommunicating segment.

For PCA exploration, posterior angulation of the probe with a depth of 55–75 mm is required; P1-precommunicating segment is normally red-coded, and P2-postcommunicating segment is blue-coded.

#### The transorbitary window

The probe is placed on the ocular eyelid. Images should be obtained with a reduced power output, with a mechanical index not exceeding 0.23, because of an increased safety risk (there are risks of capillary bleeding for values exceeding this threshold) [37]. Using color Doppler evaluation, physicians can identify the carotid siphon at an 80 mm depth, ophthalmic artery at a 40 mm depth, and a terminal ICA branch that passes over the optic nerve and supplies ocular globe circulation. Using a high-frequency linear probe, the ocular globe and the echogenic retrobulbar area can be shown and measured, with the assessment of Optic Nerve Sheath Diameter, which is a tool that can estimate ICP non-invasively.

#### The submandibular window

The submandibular window allows the assessment of the distal extracranial ICA, with the probe indicator directed anteriorly. The ICA is commonly posterolateral to the external carotid artery (both blue-coded) and, unlike the latter, lacks cervical branches and has a low-resistance velocity profile.

#### The transforaminal window

The transforaminal window enables the assessment of the posterior cerebral circulation. The transducer is positioned over the upper neck at the base of the skull, angled cephalad toward the nose, with the probe indicator oriented to the right. The anatomical reference landmark is the foramen magnum, a central hypoechoic structure. The color Doppler shows a “v-shaped” coded blue, corresponding to the intracranial segment of the vertebral arteries, which then constitute the basilar artery [36].

### Main tcd/tccd basic parameters

**Fig. 13:**
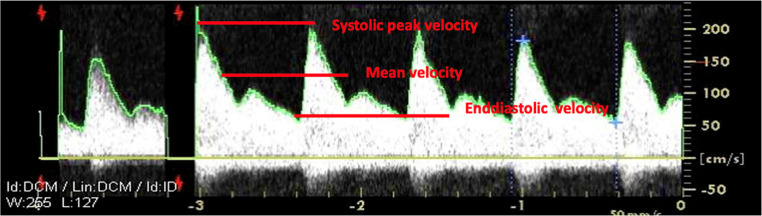
Transcranial Doppler waveform and flow velocities

**Flow velocities (**Figs. 11 and 13**and** Table 3**)**:

- Peak Systolic Velocity (SV): which corresponds to each tall “peak” in the spectrum window.
- Diastolic velocity (DV): which corresponds to the diastolic, or lower component in the spectrum window.
- End diastolic velocity (EDV): the velocity at the end of the diastole.
- Mean velocity (MV): the mean flow velocity is calculated as EDV plus one-third of the difference between PSV and EDV.

Velocities depend importantly on the insonation angle, while Doppler indices are not [36]. CBF is calculated as the product of MV and the cross-sectional area of the insonated vessel; if the cross-sectional area is normal and constant, CBF will mainly depend on MV, and this is why TCD can be considered a CBF surrogate.

### Other TCD derived indices

Gosling’s pulsatility index is defined and calculated as the difference between SV and DV, divided by MV

$$P.I. =\frac{SV-DV}{MV}$$

and the Resistive index is,

$$R.I.=\frac{SV-DV}{SV}$$

**Table 3:** TCD/TCCD features

Summary of vessels identification criteria				
Artery	Window	Depth (mm)	Direction of flow (relative to transducer)	Velocity (cm/sec); normal values
MCA	TT	45–65	Toward	46–86
MCA/ACA bifurcation	TT	60–65	Bidirectional	-
ACA	TT	60–75	Away	41–76
PCA (P1)	TT	60–75	Toward	33–64
PCA (P2)	TT	60–75	Away	33–64
PICA	TT	60–75	Toward	30–48
Ophthalmic	TO	45–60	Toward	21–49
Vertebral	TF	65–85	Away	27–55
Basilar	TF	90–120	Away	30–57

**Abbreviations**: ACA: anterior cerebral artery; BA: basilar artery; CS: carotid siphon; ICA: internal carotid artery; MCA: middle cerebral artery; MV: mean velocities; PCA: posterior cerebral artery (p1: first segment; p2: second segment); TICA: terminal internal carotid artery; VA: vertebral artery

## Pupillometry

### Pupil reflex to light

#### Definition

Pupillometry is an autonomic reflex that, in response to a light stimulus, regulates the amount of light that reaches the retina through pupil constriction. The constriction occurs through the innervation of the iris sphincter muscle, itself controlled by the parasympathetic system.

**Fig. 14:**
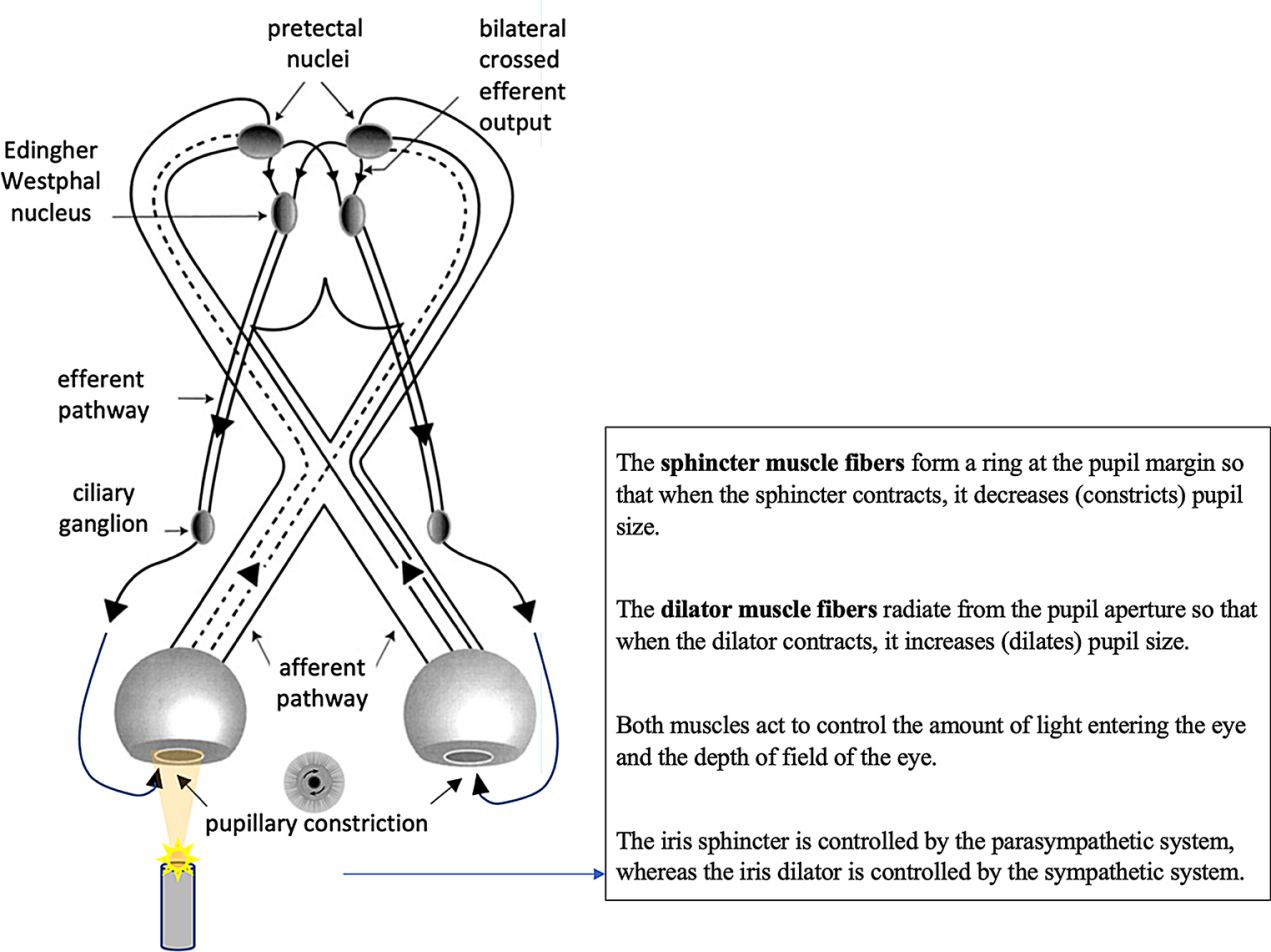
Pupillary reflex to light

The retinal ganglion cell layer gives rise to the afferent pupillary fibers that travel through the optic nerve, optic chiasm, and optic tract, join the brachium of the superior colliculus, and then travel to the pretectal area. The pretectal area sends fibers bilaterally to the efferent Edinger-Westphal nuclei of the oculomotor complex. From here, efferent pupillary parasympathetic preganglionic fibers travel along the oculomotor nerve, synapsing in the ciliary ganglion. The ciliary ganglion later sends parasympathetic postganglionic axons in the short ciliary nerve, innervating the iris sphincter smooth muscle via M3 muscarinic receptors. Bilateral innervation of the Edinger-Westphal nuclei permits a direct and consensual pupillary response to light (Fig. 14) [38].

Pupil dilation acts in opposition to parasympathetically mediated pupil constriction and is mediated by sympathetic output. Sympathetic pathways, which are inhibited by light, originate in retina-receptive neurons of the pretectum and the suprachiasmatic nucleus. Following light stimulation, the inhibition of these pathways gives rise to the light reflex, leading to pupillary constriction. Light stimulates both the noradrenergic and the serotonergic pathways.

### Non-quantitative pupillary light reflex examination

#### Definition

This is the evaluation of pupil reactivity to light, or pupillary light reflex (PLR), conventionally performed through the use of a light stimulus, natural or artificial, derived from various types of sources (penlight, smartphone, natural light, flashlight). It is a qualitative evaluation of pupil size and symmetry, reactivity, velocity, and the extent of constriction, mostly left to the interpretation of the examiner, thereby subject to low inter and intra-rater correlations [39].

The characteristics and additional definitions of pupil reactivity and constriction following direct light stimulation are listed in Table 4.

**Table 4:** Pupil reactivity and constriction following direct light stimulation. Variables which can be evaluated using non-quantitative pupillary examination

Mydriasis	The dilation of the pupil due to a physical response or a non-physiological cause.
	Non-physiological causes of mydriasis include trauma, disease, and certain drugs that affect the sympathetic (stimulation) and parasympathetic (inhibition) systems.
Miosis	Excessive pupil constriction is known as miosis, where the diameter of the pupil is less than 2 mm. Another term for miosis is a pinpoint pupil. Similar to mydriasis, miosis can result from either a physical response or a non-physiological cause. Non-physiological causes of miosis include trauma, disease, and certain drugs that affect the parasympathetic (stimulation) and sympathetic (inhibition) systems.
Sluggish reaction to light	The velocity of reactivity to light may be reduced due to direct or indirect trauma, brain disease, ischemia, increased intracranial pressure, diffuse cellular cerebral dysfunction (toxic-metabolic encephalopathy), or the use of certain drugs.
Brisk reaction	The normal, quick pupil response to light stimulation to light
Anisocoria	Pupils asymmetric in size. Physiologic anisocoria in the population is quite common. The variation should be < 1 mm, and both eyes should react to light in a normal way. May represent an underlying pathological manifestation, such as 3rd nerve damage (e.g., cerebral expansion).

### Quantitative pupillary light reflex examination (automated pupillometry)

#### Definition

An exam performed through the use of a quantitative pupillometer device, which is a non-invasive handheld device capable of emitting light, with a display screen, and performing infrared-based measurements of the pupillary light reflex.

**Fig. 15:**
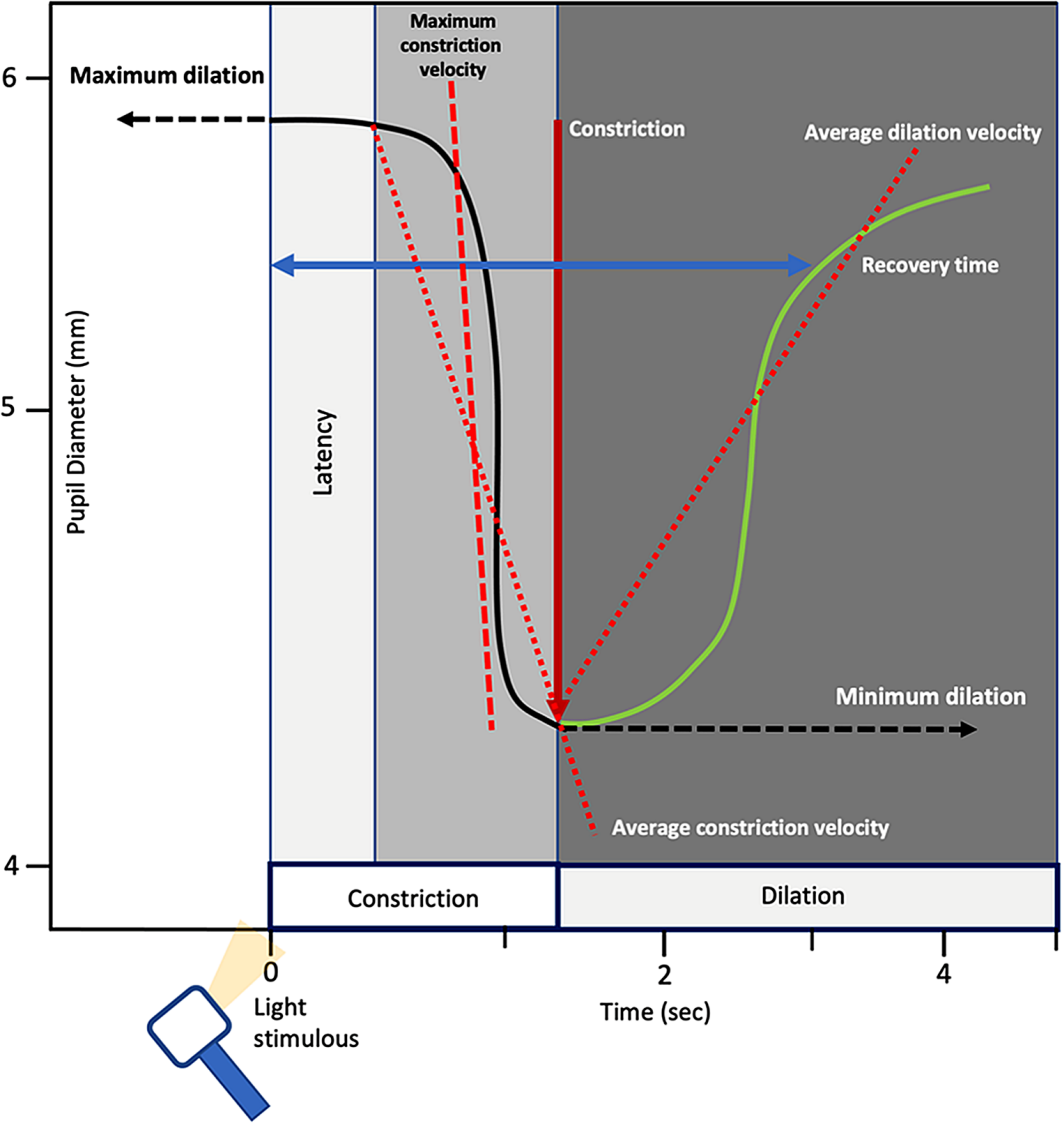
PLR phases measured by a quantitative pupillometer: Latency: onset time of constriction following a light stimulus. Constriction velocity and maximum constriction velocity, calculated as the slope during the constriction phase. Dilation velocity calculated as the slope during the dilation phase. Modified and reproduced from [40]

The PLR measurement consists of four distinct phases, which are based on variations in pupil diameter over time following light stimulation (Fig. 15).

Quantitative pupillometry provides objective parameters measured during the four phases: pupil size, latency, constriction, and dilation velocity (Table 5).

**Table 5:** Parameters measured by a quantitative pupillometer

Parameter	Definition	Normal value
Max	Pupil diameter at rest (before constriction, mm)	Asymmetry < 0.5 mm
Min	The peak pupil diameter constriction (mm)	Asymmetry < 0.5 mm
%CH	Percentage change (max–min)/size as max (%)	≥ 15%: brisk (normal) 1–14%: sluggish 0%: non-reactive
LAT	The onset time of constriction following initiation of the light stimulus (sec)	0.24–0.28 s
CV	Average measure of the pupil diameter constriction velocity (mm/sec)	≥ 1.0–1.5 mm/sec
MCV	Maximum velocity of the pupil constriction responding to the light stimulation (mm/sec)	
DV	Distance of the re-dilatation divided by the duration of the re-dilatation (mm/sec)	Up to 2.83 mm/sec

**Abbreviations**: Max, maximum; min, minimum; %CH, percentage change; LAT, latency; CV, constriction velocity; MCV, maximum CV; DV, dilatation velocity;

### The neurological pupil index (NPi; *NeurOptics*)

The NPi is calculated through an algorithm, which incorporates multiple parameters: baseline pupil diameter at baseline, % of change, latency, CVs, and DVs (Table 5).

**Fig. 16:**
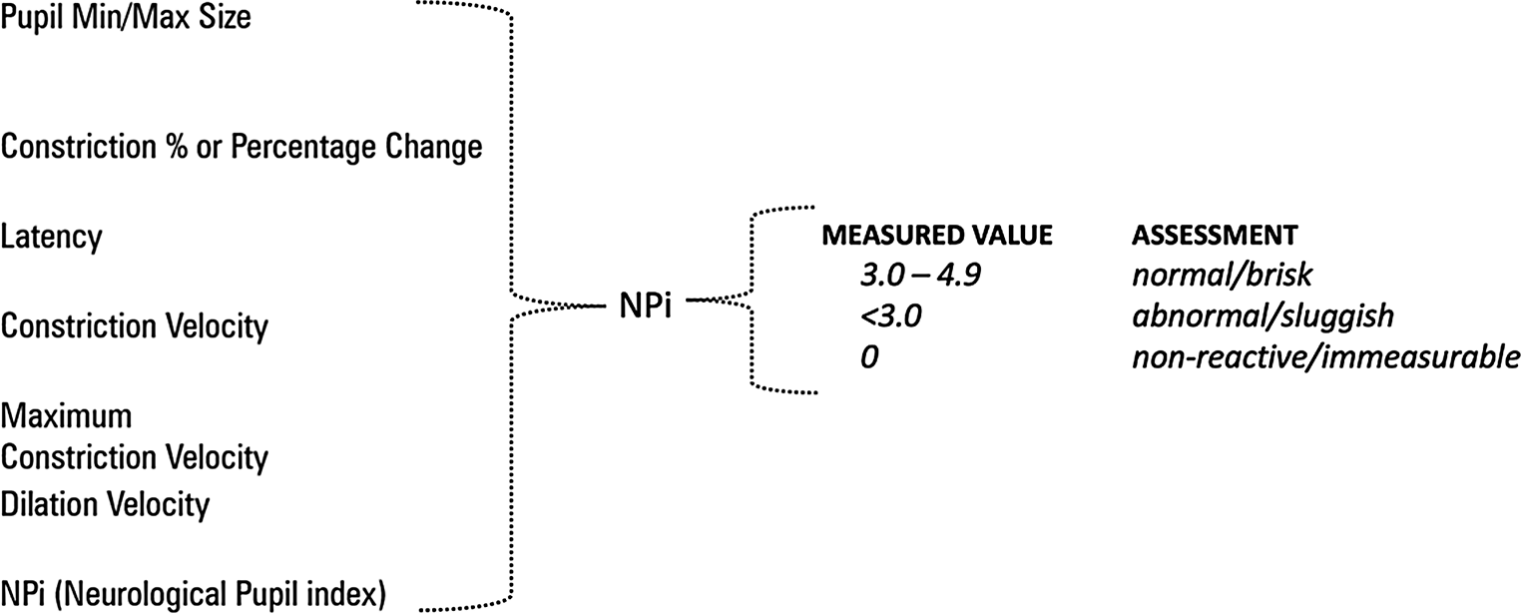
On the left are the input variables, and on the right are the reference values of NPi

A value of NPi ranging from 3 to 5 is considered normal, while an NPi < 3.0 is considered abnormal, and an NPi of 0 indicates a fixed pupil. In Fig. 16, the input variables are on the left, and the reference values of NPi are on the right.

### The Pupillary Light Reflex (PLR, *Algiscan*)

The PLR, as captured by quantitative pupillometry, serves another function related to its property of gradually increasing in response to pain stimulation [41]. After measuring the baseline PLR, both responses are assessed with electrical stimulation, employing a gradual stepwise increase in the intensity from 10 to a maximum of 60 mA. The stimulation is applied to the left forearm by connecting two electrodes to the pupillometer. The following parameters are measured: pupil diameter (mm), pupillary reflex dilation (PRD) to pain (%), and the pupillary pain index (PPI). PPI is calculated after stopping electrical stimulation when the PRD exceeds 13% during stimulation. A PPI score of 1 indicates that the PRD is below 5% during stimulation at maximal intensity (60 mA), while a PPI of 9 indicates that the PRD is above 13% during stimulation at 10 mA. A PPI score < 4 is typically considered adequate for pain control [39, 40].

## Conclusions

Patients undergoing major surgery and those admitted to neurosurgical and non-neurosurgical ICUs are frequently affected by multi-organ failure. The main reason for ICU admission could be related to primary organ damage (e.g., respiratory insufficiency due to pneumonia, myocardial infarction, acute kidney injury), but due to the inevitable organ crosstalk process, many other organs are eventually involved. Nowadays, the brain is the least investigated organ when compared to all others, even though, directly or indirectly, its physiology and function are variably compromised. Huge efforts have been made in the past to monitor organ function to improve therapeutic approaches (e.g., ventilator mode in relation to respiratory pressures, inotropes/vasoactive drugs depending on US-based cardiac function, fluid therapy relative to US lung investigation). Investigations of brain function have mostly been limited to neuroanesthesia and neurosurgical settings. Today, new non-invasive technologies make it possible to bring neurological investigative tools into settings that are not primarily neurological. EEG, pEEG, cEEG, qEEG, rSO2, transcranial Doppler, and pupillometry can help clinicians diagnose and monitor the functional condition of the central nervous system of any patient undergoing anesthesia or admitted to the ICU. The time has come to consider neurological monitoring as a standard of care to be applied to our patients regardless of the environment in which they are admitted. In medicine, as in finance, *“If you can’t measure it, you can’t manage it”* (Peter Drucker, Businessman). Finally, we should always remember that it is not the monitoring itself that improves patients’ outcomes, but the therapies guided by the information we derive, as it can change the clinical course.

### Electronic supplementary material

Below is the link to the electronic supplementary material.

Supplementary Material 1

Supplementary Material 2

## Abbreviations

AEEG: amplitude electroencephalography
ASYM: asymmetry
BS: burst suppression
BSR: burst suppression ratio
CA: cerebral autoregulation
CBF: V-cerebral blood flow velocity
CDSA: color density spectral array
cEEG: continuous electroencephalography
CPP: cerebral perfusion pressure
CSA: color spectral array
CV: constriction velocity
DCA: delayed cerebral ischemia
DPF: differential pathlength factor
DSA: density spectral array
ελ: extinction coefficient of a chromophore
EMG: electromyography
EMR: absorb electromagnetic radiation
FFT: fast Fourier transform
HHB: deoxyhemoglobin
Hz: Hertz
ACA: anterior cerebral artery
DV: diastolic velocity
EDV: end diastolic velocity
ICA: internal carotid artery
ICH: intracranial hemorrhage
ICP: intracranial pressure
ICU: intensive care unit
LED: light-emitting diode
MCA: middle cerebral artery
MCV: maximum constriction velocity
MV: mean velocity
NCS: nonconvulsive seizures
nCPP: non-invasive cerebral perfusion pressure
NIRS: near infrared spectroscopy
nPI: neurological pupil index
OD: optical density
O_2_Hb: oxyhemoglobin
OR: operating room
PaCO_2_: partial pressure of carbon dioxide
PCA: posterior cerebral artery
pEEG: processed electroencephalography
qEEG: quantitative electroencephalography
PD: pulsed Doppler
PLR: pupillary light reflex
RSE: refractory status epilepticus
rSO_2_: regional oxygen saturation
SaO_2_: arterial Hb oxygen saturation
SAH: subarachnoid hemorrhage
SEF_95_L: spectral edge frequency 95% left side
SEF_95_R: spectral edge frequency 95% right side
SV: Peak Systolic Velocity
TBI: traumatic brain injury
TCD: transcranial Doppler
TCCD: transcranial color-duplex Doppler
tHB: total hemoglobin
