# Supplementary material for: Non-invasive technology for brain monitoring: definition and meaning of the principal parameters for the International PRactice On TEChnology neuro-moniToring group (I-PROTECT)
Source: J Clin Monit Comput. 2024 Mar 21;38(4):827–45. doi: 10.1007/s10877-024-01146-1 (PMC11297817; doi:10.1007/s10877-024-01146-1)
Supplement: Supplementary file 2 — Supplementary Material 2 [file 10877_2024_1146_MOESM2_ESM.docx]

**Transcranial Doppler and Transcranial Color-Coded Duplex Doppler Sonography: clinical utility and applications: vasospasm, intracranial pressure and cerebral perfusion pressure estimation, brain death, cerebral autoregulation investigation.**

**VASOSPASM**

**Definition:** Vasospasm is the narrowing of cerebral vessels after subarachnoid hemorrhage, resulting in reduced CBF (Figure 1) [1]. Digital subtraction angiography is the gold standard examination for diagnosis of cerebral vasospasm, but the use of TCD and TCCD has a fundamental role in the bedside monitoring of these patients [2–4]. Guidelines for the management of aneurysmal subarachnoid hemorrhage are recommended with class IIa monitoring of vasospasm by TCD [2,4]**.**

- Definition of vasospasm: A flow velocity increase of > 25-40 cm/s and/or a 50% increase compared to baseline conditions in 24 hours is considered an alarm for the development of vasospasm.
- Vasospasm in MCA can be classified into mild, moderate, and severe based on the flow velocity increases of 80–120 cm/s, 120–200 cm/s, >200 cm/s, respectively, while a blood flow velocity > 85 cm/s is defined as threshold for the diagnosis of vasospasm of the basilar artery [5].

**Figure 1**. Cerebral vasospasm is demonstrated through digital subtraction angiography and confirmed at TCD insonation as increased flow velocities (mean flow velocity = 190 cm/sec)


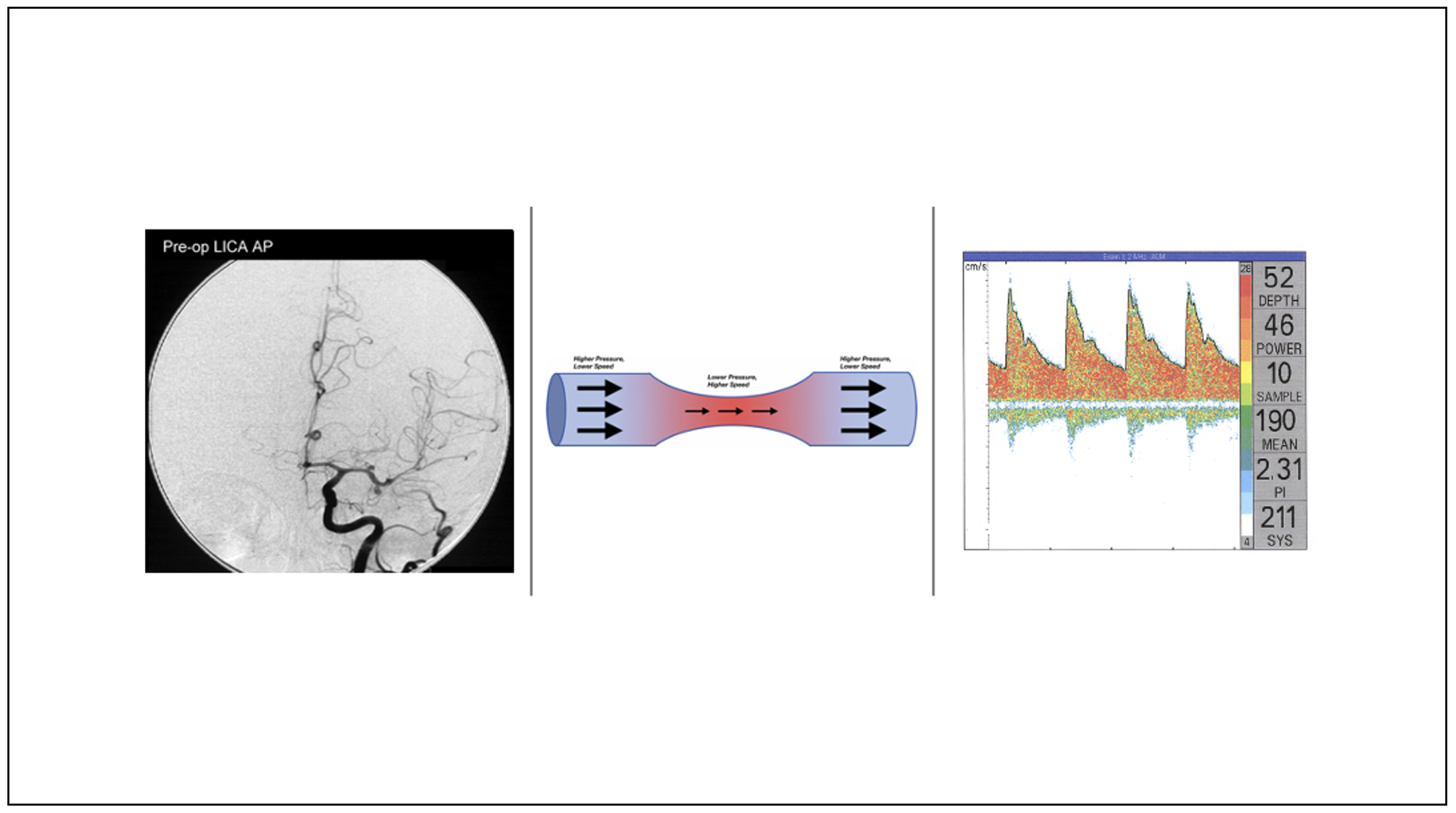


Increased flow velocities in cerebral vessels occur not only in cases of vasospasm, but also in the presence of other causes: hyperemia, increased PaCO_2_ values, loss of autoregulation, arteriovenous malformations. Therefore, it is important to integrate the ultrasound examination with the assessment of the Lindegaard ratio.

- Lindegaard ratio is defined as the ratio of the mean FV of the MCA to mean FV of the extracranial portion of the ipsilateral ICA. A value greater than 3 indicates vasospasm; values less than 3 indicate hyperemia.
- A modified Lindegaard ratio (Sviri ratio) has been proposed for the vasospasm of the basilar artery, and it is calculated as the ratio between the mean FV in basilar and vertebral artery [6]. A mean velocity above 85 cm/sec and a Sviri ratio > 3 are suggestive of vasospasm (50% occlusion) with a sensitivity of 92% and specificity of 97% [7] (Table 1).

| Vasospasm | Mean flow velocity (cm/s) | Lindegaard Index or Sviri ratio |
| --- | --- | --- |
| Middle Cerebral Artery | | |
| Mild | 80–120 | 3–4 |
| Moderate | 120–200 | 4–5 |
| Severe | >120 | >5 |
| Basilar Artery Velocity | | |
| Mild | 70–80 | >2 |
| Moderate | >85 | 2.5–3 |
| Severe | >85 | >3 |

Table 1: Criteria for vasospasm, Lindegaard ratio and Sviri ratio.

Intracranial pressure and cerebral perfusion pressure estimation by trancranial Doppler and transcranial color Doppler

Elevated ICP is defined as increased values > 22 mmHg, and can cause the development of diffuse swelling and herniation [8].

Monitoring and optimization of both CPP and ICP play a central role in severe traumatic brain injury, hemorrhagic stroke (SAH, intraparenchimal bleeding), ischemic stroke, hydrocephalus, hepatic encephalopathy, and alterations in venous outflow.

TCD and TCCD assessment of ICP include (Figure 2),

- Analysis of cerebral blood flow velocity waveform
- Optic nerve sheath diameter measurement

**Figure 2**. Waveform analysis and flow velocity of the middle cerebral artery and optic nerve sheath diameter in a patient with increased intracranial pressure


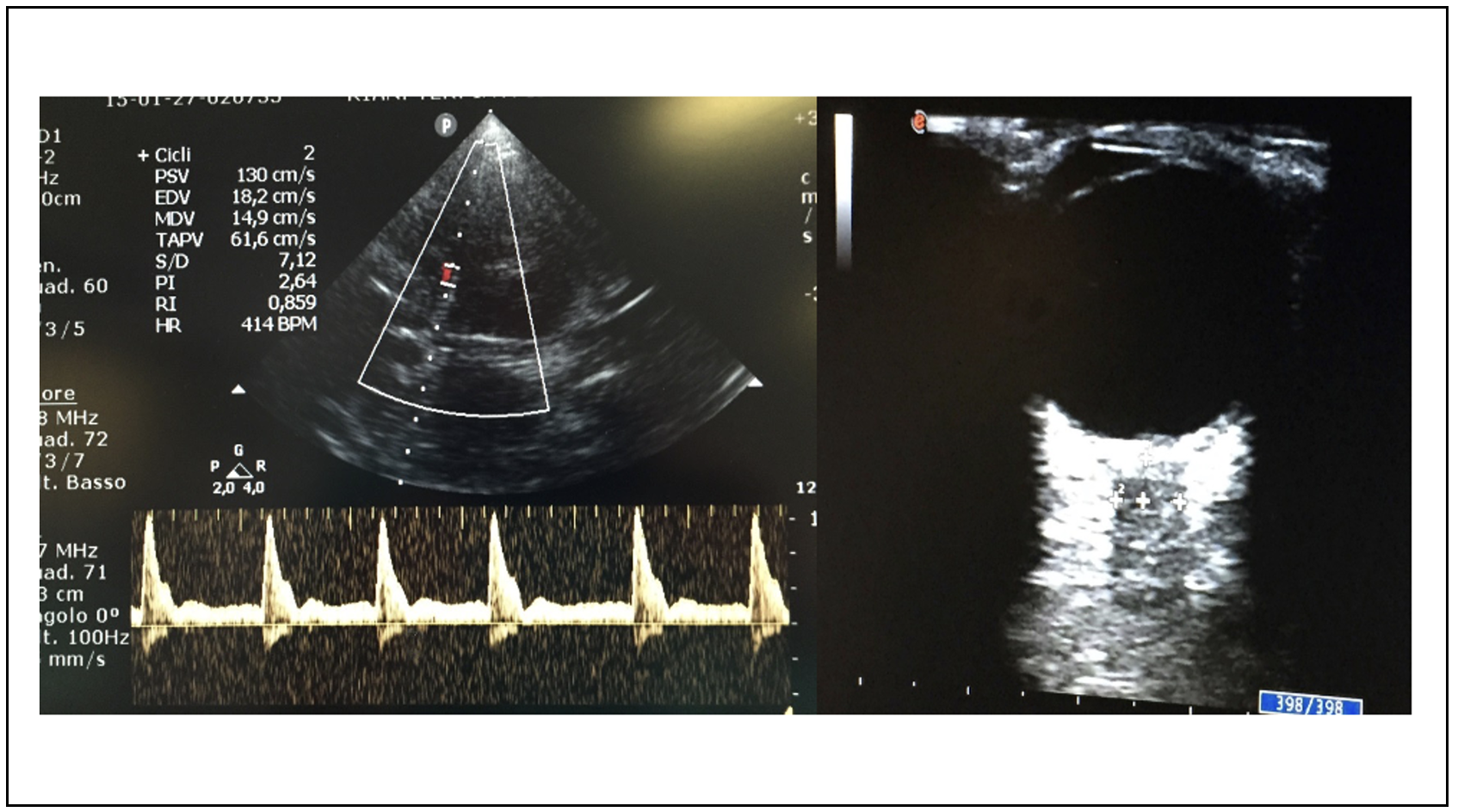


**Analysis of cerebral blood flow velocity waveform assessed by transcranial Doppler or transcranial color Doppler**

When assessing CBFV with TCD, increased ICP causes a drop in the diastolic FV and increase in the Pulsatility Index (PI) [9,10].

TCD-derived methods for calculating non-invasive ICP can be classified into the following 3 categories:

1. methods based on the TCD-derived PI,
2. methods based on calculation of noninvasive cerebral perfusion pressure,
3. methods based on mathematical models associating cerebral blood flow velocity and arterial blood pressure.
4. **Methods based on the TCD-derived PI**

The Gosling PI is a ratio derived from the difference between the systolic and diastolic FV (FVd) over mean FV.

The effect of the increase in ICP results in a reduction in diastolic FV (earlier than changes in systolic flow velocity (FVs)), with a consequent increase in PI. However, the use of this parameter is controversial, as the value depends not only on the change in resistance (increase in ICP), but also on CPP, systemic blood pressure, and changes in PaCO_2_. A formula was proposed to transform the PI value into a ICP value (sensitivity 89% and specificity 92%) [11].

$$ICP=\left( 10.93\times PI \right)-1.28$$

According to this formula, a value of PI of 2.13 should be the cut off for intracranial hypertension as it yields an ICP value of 22 mmHg or more; nevertheless, PI < 1.2 is usually considered normal.

### **Methods based on the calculation of non-invasive CPP (nCPP)**

Mathematical formulas have been proposed to estimate CPP (nCPP). Calculating CPP, it is possible to estimate ICP according to the following relationship:

$$ICP=MAP-nCPP$$

Aaslid *et al.* proposed this approach, suggesting a formula for the estimation of CPP based on the spectral PI and the first harmonic component of the ABP. This formula demonstrated to be quite sensitive to the variation of CPP but showed important limitation in accuracy [6].

$$nCPP=FVm\times\frac{A}{f}$$

Czosnyka M. *et al*. used the following formula [12,13].

$$nCPP=MAP\times\left( \frac{FVd}{FVm} \right)+14$$

The value of arterial blood pressure (ABP) below which collapses and cessation of blood flow occurs is defined as critical closing pressure (CrCP). CrCP is equal to the sum of ICP with vascular wall tension (WT). In view of this association, the following formula for estimating nCPP has been proposed [13]:

$nCPP=ABP \times\left\lfloor0.734- \frac{0.266}{\sqrt{{1+ \left( \frac{CPP}{FV} \times\frac{CaBV1}{a1}\times HR \times2\pi\right)}^{2}}} \right\rfloor-7$.26

**Optic nerve sheath diameter**

Increased ICP is transmitted to the subarachnoid compartment of the nerve, thus potentially causing enlargement of the optic nerve sheath diameter (Figure 2).

Validation of this method has been achieved by comparison with invasive ICP measurements. In adult patients with signs of intracranial hypertension on CT, the optic nerve sheath diameter value ranged between 4.84 and 6.4 mm; in contrast, in patients without radiological signs of intracranial hypertension, measurements ranged between 3.49 and 4.94 mm, with good sensitivity and specificity [14]. Measurements by optic nerve sheath diameter showed good accuracy (0.811–0.954) for the diagnosis of intracranial hypertension, with a ROC curve of 0.938 [15–18]. A new meta-analysis published in 2022 that included 619 patients found that sensitivity was 90% (95% CI: 85%-94%] and specificity was 85% (95% CI: 80%-89%) [18].

Despite the results of these studies, the diagnostic cutoff for intracranial hypertension is still much debated, with many studies reporting an optimal cutoff ranging from 5 to 6 mm.

**Brain death evaluation with TCD/TCCD**

Growing intracranial hypertension is associated with progressive reduction in diastolic flow velocity. When ICP equals systemic diastolic pressure, the disappearance of the diastolic flow velocity is observed. A further increase in ICP leads to the appearance of reverberant flow (negative diastolic flow velocity), until the appearance of systolic spikes (systolic flow velocity peaks of low amplitude and are present only in the proto-systolic phase), followed by the complete disappearance of the Doppler signal (Figure 3).

**Figure 3**. Doppler signal during cerebral circulatory arrest


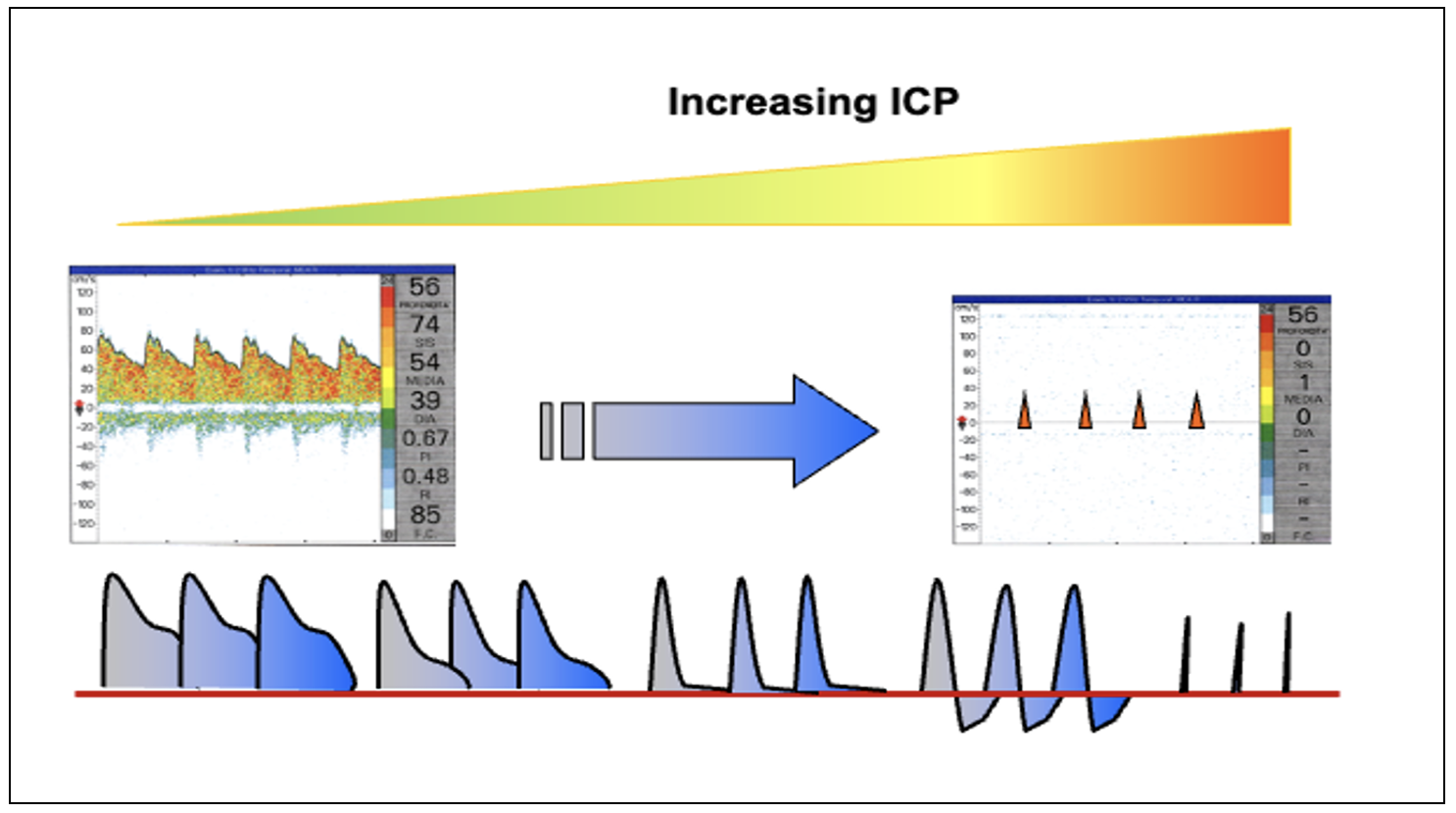


A recent meta-analysis explored the accuracy of TCD for the diagnosis brain death (BD) suggested a pooled sensitivity and specificity estimates of 90% (95% CI, 87%–0.92%) and 98% (95% CI, 96%–99%), respectively. This suggests that TCD is a highly accurate ancillary test in the context of suspected BD [19].

**Cerebral autoregulation**

**Definition:** Cerebral autoregulation (CA) is the ability of the brain to maintain blood flow constantly, despite the changes in cerebral perfusion pressure (within a range of 50–150 mmHg in healthy subjects).

TCD allows both testing and monitoring several components of CA by application of different techniques.

Autoregulation of the cerebral circulation can be tested by examining the changes in FV in response to the changes in PaCO_2_ and MAP.

“Test” techniques comprise the following:

- Aaslid ‘cuff test’ method. According to this method, arterial hypotension is induced by placing thigh cuffs on each thigh increasing pressure 50mmHg above the PAS for 3 min and then quickly releasing them, all this while measuring MCA flow velocity using TCD [20]. This transition allows calculation of both the dynamic rate of regulation, dRoR, whose normal value is 20%/sec (percentage per second); this indicates how quickly the velocity of cerebral flow returns to its starting level after the hypotensive stimulus, and of the index of dynamic autoregulation. Dynamic ARI is a dimensionless index ranging from 0 to 9, which describes the response of CBF to a steep decrease in ABP; the threshold value for a preserved autoregulation is around 5. However, inducing rapid changes in systemic arterial pressure in patients who are already seriously compromised could be harmful, so this limits the application of the cuff test.
- Transient hyperemic response test (Figure 4). This method is based on the compensatory vasodilatation of the cerebral distal arterioles occurring after a brief compression of the common carotid artery. The test involves measuring the systolic speed of flow in the MCA at basal conditions and then the ipsilateral common carotid is compressed for 5–8 s, causing a reduction in CPP.
- If autoregulation is intact, during CPP reduction the cerebral arterioles respond through vasodilatation, in order to reduce resistance and maintain the CBF constant. When compression is released, this causes a temporary increase in blood flow as CPP acts on a dilated vascular bed. The THRR, is defined as the ratio between the velocity of systolic flow during the hyperemic phase (two cycles after the compression release excluding the very first cycle) and the velocity of basic systolic flow (five cycles before compression). Normal values range between 1.105 and 1.29 (average (95% CI) 1.2 (1.17–1.24)), with a threshold of 1.10 as the lower limit for a normal response[21].

Continuous monitoring of cerebral autoregulatory function (Figure 14) entails the availability of a dedicated TCD machine for monitoring sessions and a dedicated software for FV wave and CPP or ABP signals collection and analysis. The “mean flow velocity index” (Mx index) is calculated as the correlation (coefficient) between CPP and TCD detected mean flow velocity in the MCA: a positive correlation suggests an absence of vasomotor response and therefore poor autoregulatory capacity, whereas a negative correlation indicates preserved autoregulatory function. Mxa is the correlation coefficient between ABP and FVIn patients with TBI, negative values or values < 0.3 indicate intact autoregulation, whereas values > 0.3 failure of cerebral autoregulation [22–24].

**Figure 4**. Static and dynamic methods to assess cerebral autoregulation. THRR: transient hyperemic response ratio; ratio between the velocity of systolic flow during the hyperemic phase (two cycles after the compression release excluding the very first cycle) and the velocity of basic systolic flow (five cycles before compression).


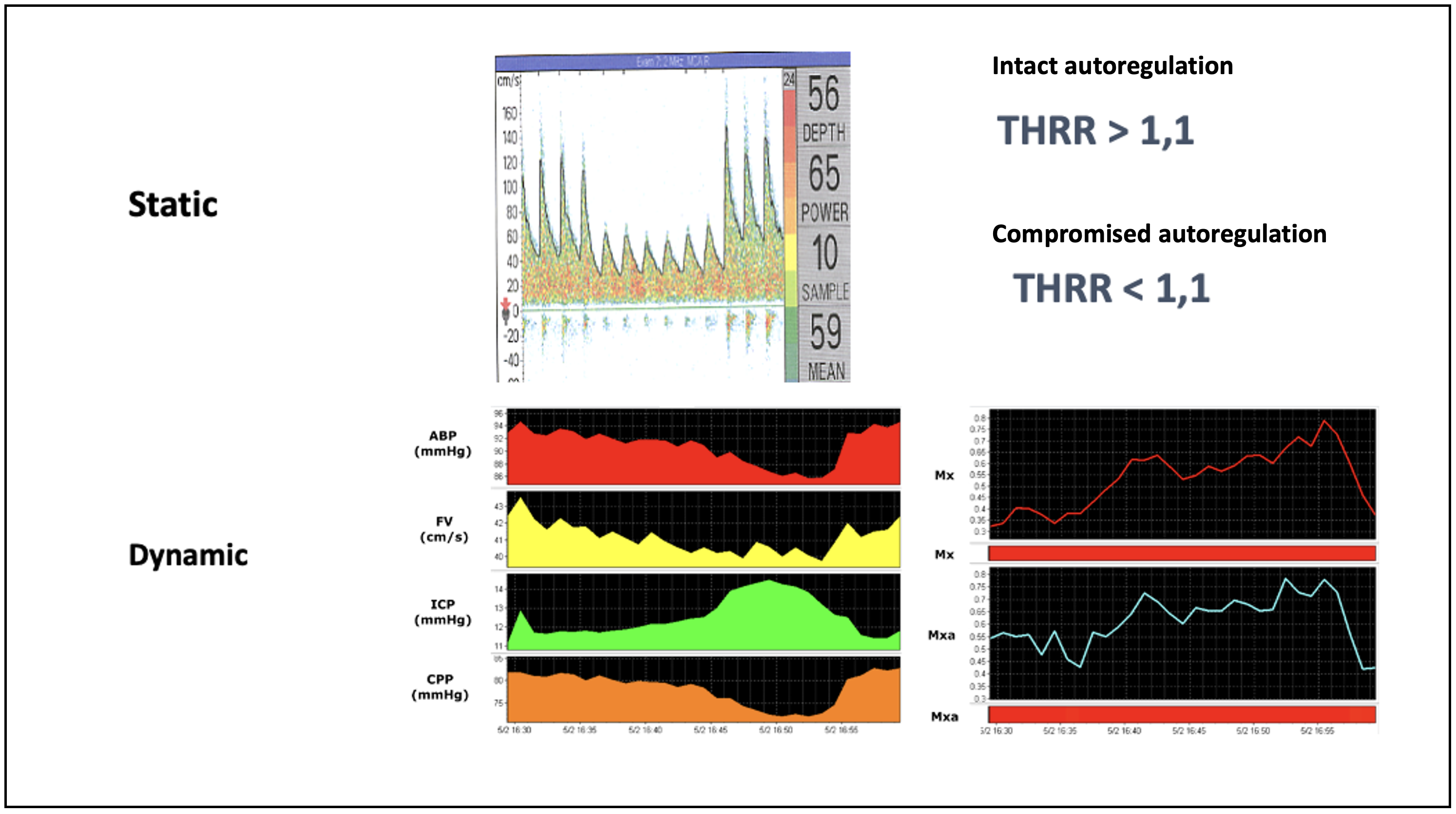


**References**

1. Connolly ES, Rabinstein AA, Carhuapoma JR, Derdeyn CP, Dion J, Higashida RT, et al. Guidelines for the management of aneurysmal subarachnoid hemorrhage: a guideline for healthcare professionals from the American Heart Association/american Stroke Association. Stroke. Stroke; 2012;43:1711–37.

2. Rasulo FA, De Peri E, Lavinio A. Transcranial Doppler ultrasonography in intensive care. Eur J Anaesthesiol. 2008;25:167–73.

3. Li K, Barras CD, Chandra R V., Kok HK, Maingard JT, Carter NS, et al. A Review of the Management of Cerebral Vasospasm After Aneurysmal Subarachnoid Hemorrhage. World Neurosurg [Internet]. Elsevier Inc; 2019;126:513–27. Available from: https://doi.org/10.1016/j.wneu.2019.03.083

4. Aaslid R. Transcranial Doppler assessment of cerebral vasospasm. Eur J Ultrasound. 2002;16:3–10.

5. Washington CW, Zipfel GJ. Detection and monitoring of vasospasm and delayed cerebral ischemia: A review and assessment of the literature. Neurocrit Care. 2011;15:312–7.

6. Aaslid R, Lundar T, Lindegaard KF, Nornes H. Estimation of Cerebral Perfusion Pressure from Arterial Blood Pressure and Transcranial Doppler Recordings. Intracranial Press VI. Springer, Berlin, Heidelberg; 1986;226–9.

7. Sviri GE, Ghodke B, Britz GW, Douville CM, Haynor DR, Mesiwala AH, et al. Transcranial Doppler grading criteria for basilar artery vasospasm. Neurosurgery. Neurosurgery; 2006;59:360–5.

8. Dostovic Z, Dostovic E, Smajlovic D, Ibrahimagic OC, Avdic L. Brain Edema After Ischaemic Stroke. Med Arch (Sarajevo, Bosnia Herzegovina). 2016;70:339–41.

9. Czosnyka M, Richards HK, Whitehouse HE, Pickard JD. Relationship between transcranial Doppler-determined pulsatility index and cerebrovascular resistance: An experimental study. J Neurosurg. 1996;84:79–84.

10. Bellner J, Romner B, Reinstrup P, Kristiansson KA, Ryding E, Brandt L. Transcranial Doppler sonography pulsatility index (PI) reflects intracranial pressure (ICP). Surg Neurol. 2004;62:45–51.

11. Cardim D, Robba C, Bohdanowicz M, Donnelly J, Cabella B, Liu X, et al. Non-invasive Monitoring of Intracranial Pressure Using Transcranial Doppler Ultrasonography: Is It Possible? Neurocrit Care. 2016;25:473–91.

12. Rasulo FA, Calza S, Robba C, Taccone FS, Biasucci DG, Badenes R, et al. Transcranial Doppler as a screening test to exclude intracranial hypertension in brain-injured patients: the IMPRESSIT-2 prospective multicenter international study. Crit Care [Internet]. BioMed Central; 2022;26:1–13. Available from: https://doi.org/10.1186/s13054-022-03978-2

13. Varsos G V., Kolias AG, Smielewski P, Brady KM, Varsos VG, Hutchinson PJ, et al. A noninvasive estimation of cerebral perfusion pressure using critical closing pressure. J Neurosurg. J Neurosurg; 2015;123:638–48.

14. Rasulo FA, Bertuetti R. Transcranial Doppler and Optic Nerve Sonography. J Cardiothorac Vasc Anesth. 2019;33 Suppl 1:S38–52.

15. Robba C, Santori G, Czosnyka M, Corradi F, Bragazzi N, Padayachy L, et al. Optic nerve sheath diameter measured sonographically as non-invasive estimator of intracranial pressure: a systematic review and meta-analysis. Intensive Care Med [Internet]. Springer Berlin Heidelberg; 2018;44:1284–94. Available from: https://doi.org/10.1007/s00134-018-5305-7

16. Aletreby W, Alharthy A, Brindley PG, Kutsogiannis DJ, Faqihi F, Alzayer W, et al. Optic Nerve Sheath Diameter Ultrasound for Raised Intracranial Pressure: A Literature Review and Meta-analysis of its Diagnostic Accuracy. J Ultrasound Med. 2022;41:585–95.

17. Wu GB, Tian J, Liu XB, Wang ZY, Guo JY. Can optic nerve sheath diameter assessment be used as a non-invasive tool to dynamically monitor intracranial pressure? J Integr Neurosci. 2022;21.

18. Cardim D, Griesdale DE, Ainslie PN, Robba C, Calviello L, Czosnyka M, et al. A comparison of non-invasive versus invasive measures of intracranial pressure in hypoxic ischaemic brain injury after cardiac arrest. Resuscitation [Internet]. European Resuscitation Council, American Heart Association, Inc., and International Liaison Committee on Resuscitation.~Published by Elsevier Ireland Ltd; 2019;137:221–8. Available from: https://doi.org/10.1016/j.resuscitation.2019.01.002

19. Chang JJ, Tsivgoulis G, Katsanos AH, Malkoff MD, Alexandrov A V. Diagnostic accuracy of transcranial doppler for brain death confirmation: Systematic review and meta-analysis. Am J Neuroradiol. 2016;37:408–14.

20. Giller CA. A bedside test for cerebral autoregulation using transcranial Doppler ultrasound. Acta Neurochir (Wien). Acta Neurochir (Wien); 1991;108:7–14.

21. Cavill G, Simpson EJ, Mahajan RP. Factors affecting assessment of cerebral autoregulation using the transient hyperaemic response test. Br J Anaesth. 1998;81:317–21.

22. Czosnyka M, Smielewski P, Lavinio A, Pickard JD, Panerai R. An assessment of dynamic autoregulation from spontaneous fluctuations of cerebral blood flow velocity: A comparison of two models, index of autoregulation and mean flow index. Anesth Analg. 2008;106:234–9.

23. Budohoski KP, Czosnyka M, De Riva N, Smielewski P, Pickard JD, Menon DK, et al. The relationship between cerebral blood flow autoregulation and cerebrovascular pressure reactivity after traumatic brain injury. Neurosurgery. 2012;71:652–60.

24. Czosnyka M, Brady K, Reinhard M, Smielewski P, Steiner LA. Monitoring of cerebrovascular autoregulation: Facts, myths, and missing links. Neurocrit Care. 2009;10:373–86.
