# Supplementary material for: Non-invasive technology for brain monitoring: definition and meaning of the principal parameters for the International PRactice On TEChnology neuro-moniToring group (I-PROTECT)
Source: J Clin Monit Comput. 2024 Mar 21;38(4):827–45. doi: 10.1007/s10877-024-01146-1 (PMC11297817; doi:10.1007/s10877-024-01146-1)
Supplement: Supplementary file 1 — Supplementary Material 1 [file 10877_2024_1146_MOESM1_ESM.docx]

To: Prof. Moritz Flick,

Deputy Editor-in-Chief

Journal of Clinical Monitoring and Computing

**RE: Non-invasive technology for brain function monitoring: definition and meaning of the principal parameters.**

For the *International PRactice On TEchnology neuro-monitoring group* (*I-PROTECT*).

**Stefano Romagnoli, M.D., Ph.D.**

Dear Professor Flick,

dear editor,

We have revised the manuscript again taking into account all the excellent suggestions of the reviewers. I would like to thank them for the invaluable help in improving the manuscript.

Uploaded you can find the following files:

- Text_revision_3_05_FEB;
- Appendix TCD_2 (not modified)
- Cover letter.

Thank you for the important opportunity.

On behalf of all the authors

Stefano Romagnoli

Sincerely,

Stefano Romagnoli, M.D., Ph.D

[stefano.romagnoli@unifi.it](mailto:stefano.romagnoli@unifi.it)
